# End-of-Life Care Preferences of Older Patients with Multimorbidity: A Mixed Methods Systematic Review

**DOI:** 10.3390/jcm10010091

**Published:** 2020-12-29

**Authors:** Ana I. González-González, Christine Schmucker, Julia Nothacker, Edris Nury, Truc Sophia Dinh, Maria-Sophie Brueckle, Jeanet W. Blom, Marjan van den Akker, Kristian Röttger, Odette Wegwarth, Tammy Hoffmann, Ferdinand M. Gerlach, Sharon E. Straus, Joerg J. Meerpohl, Christiane Muth

**Affiliations:** 1 Institute of General Practice, Goethe University, 60590 Frankfurt am Main, Germany; dinh@allgemeinmedizin.uni-frankfurt.de (T.S.D.); brueckle@allgemeinmedizin.uni-frankfurt.de (M.-S.B.); m.vandenAkker@allgemeinmedizin.uni-frankfurt.de (M.v.d.A.); gerlach@allgemeinmedizin.uni-frankfurt.de (F.M.G.); muth@allgemeinmedizin.uni-frankfurt.de (C.M.); 2 Red de Investigación en Servicios de Salud en Enfermedades Crónicas (REDISSEC), 28035 Madrid, Spain; 3 Institute for Evidence in Medicine (for Cochrane Germany Foundation), Medical Center-University of Freiburg, Faculty of Medicine, University of Freiburg, 79110 Freiburg, Germany; schmucker@ifem.uni-freiburg.de (C.S.); nothacker@cochrane.de (J.N.); nury@ifem.uni-freiburg.de (E.N.); meerpohl@ifem.uni-freiburg.de (J.J.M.); 4 Department of Public Health and Primary Care, Leiden University Medical Center, 2300 RC Leiden, The Netherlands; J.W.Blom@lumc.nl; 5 Department of Family Medicine, School CAPHRI, Maastricht University, 6200 Maastricht, The Netherlands; 6 Academic Center for General Practice, Department of Public Health and Primary Care, KU Leuven, 3000 Leuven, Belgium; 7 Patient Representative, Federal Joint Committee “Gemeinsamer Bundseausschuss”, 10587 Berlin, Germany; kristianroettger@aol.de; 8 Center for Adaptive Rationality, Max Planck-Institute for Human Development, 14195 Berlin, Germany; wegwarth@mpib-berlin.mpg.de; 9 Institute for Evidence-Based Healthcare, Faculty of Health Sciences and Medicine, Bond University, Robina, QLD 4226, Australia; thoffman@bond.edu.au; 10 Department of Medicine, University of Toronto, Toronto, ON M5S 1A1, Canada; sharon.straus@utoronto.ca; 11 Cochrane Germany, Cochrane Germany Foundation, 79110 Freiburg, Germany; 12 Department of General Practice and Family Medicine, Medical Faculty OWL, University of Bielefeld, 33615 Bielefeld, Germany

**Keywords:** end of life care, patient preferences, multimorbidity, elderly, patient centered care

## Abstract

Unpredictable disease trajectories make early clarification of end-of-life (EoL) care preferences in older patients with multimorbidity advisable. This mixed methods systematic review synthesizes studies and assesses such preferences. Two independent reviewers screened title/abstracts/full texts in seven databases, extracted data and used the Mixed Methods Appraisal Tool to assess risk of bias (RoB). We synthesized findings from 22 studies (3243 patients) narratively and, where possible, quantitatively. Nineteen studies assessed willingness to receive life-sustaining treatments (LSTs), six, the preferred place of care, and eight, preferences regarding shared decision-making processes. When unspecified, 21% of patients in four studies preferred any LST option. In three studies, fewer patients chose LST when faced with death and deteriorating health, and more when treatment promised life extension. In 13 studies, 67% and 48% of patients respectively were willing to receive cardiopulmonary resuscitation and mechanical ventilation, but willingness decreased with deteriorating health. Further, 52% of patients from three studies wished to die at home. Seven studies showed that unless incapacitated, most patients prefer to decide on their EoL care themselves. High non-response rates meant RoB was high in most studies. Knowledge of EoL care preferences of older patients with multimorbidity increases the chance such care will be provided.

## 1. Introduction

End-of-life (EoL) care is generally defined as the care provided to patients in the last year of life, although for some conditions, it may last for several years [1]. Patients living with multimorbidity (i.e., two or more concomitant chronic conditions) and life-limiting chronic diseases, as well as their loved ones and health professionals, are often confronted with decisions concerning EoL care [2]. The unpredictable trajectories of disease in older patients with multimorbidity make it particularly important that patients and providers discuss EoL care preferences at an early stage [3].

Advance care planning (ACP) describes a voluntary and ongoing discussion of EoL care preferences by patients, their families and health professionals [4]. Studies have shown the benefits to patients and their caregivers of such discussions [5,6]. A randomized controlled trial showed that EoL wishes of patients receiving ACP as an intervention were much more likely to be carried out than in the control group [5]. Moreover, ACP reduces stress, anxiety and depression in family members of deceased patients [5]. Comprehensive, timely, and compassionate planning of personalized care and support that includes a discussion of EoL care should therefore be available to those who require and desire it [1].

Predicting patients’ EoL care preferences is often difficult [7], which can limit the patient-orientated provision of EoL care [2]. Patient-provider discussions of EoL care should not only ensure that patients understand their illnesses, prognoses, and the process of dying but should also elicit patients’ preferred interventions and where they would like EoL care to be administered. At some point, health professionals will also have to address the illnesses’ possible trajectories, patients’ willingness to receive supportive (i.e., potentially life-sustaining) treatment and/or palliative care (i.e., conservative care aimed at providing comfort and maintaining quality of life).

To provide decision support to health professionals and help this complex patient population in an emotionally difficult situation, we systematically reviewed EoL care preferences in older patients with multimorbidity. We based the review on a knowledge cluster of EoL care preferences that we previously identified for an evidence map we developed on health-related preferences in older patients with multimorbidity [8].

## 2. Materials and Methods

We have described the methodology in more detail in a study protocol [9] and registered the systematic review in PROSPERO (registration no. CRD42020151862).

The present manuscript follows the Preferred Reporting System Items for Systematic Review and Meta-Analysis (PRISMA) checklist [10] (see Supplementary Table S1).

### 2.1. Design

We conducted a mixed methods systematic review using the convergent integrated approach to transform data in such a way that quantitative and qualitative data could be combined, and quantitative and qualitative studies synthesized, simultaneously [11].

### 2.2. Eligibility Criteria

#### 2.2.1. Types of Study

We included primary studies that used quantitative (e.g., structured questionnaires), qualitative (e.g., interviews, focus groups) and mixed methods methodologies. We excluded case reports and articles such as conference abstracts, narrative reviews, and editorials.

#### 2.2.2. Participants

We included older patients (mean or median age ≥ 60 years) with multimorbidity (i.e., two or more simultaneous chronic conditions [12,13]). Studies focusing on patients with one chronic disease were included when authors had reported on at least one additional chronic condition in the majority (80%) of the study population. We excluded studies that only addressed the preferences of caregivers, family members, and health care professionals. We also excluded studies involving the broader public.

#### 2.2.3. Phenomenon of Interest

EoL care preferences covered (i) willingness to receive life-sustaining treatments (LSTs), (ii) willingness to opt for the palliation of symptoms, (iii) the place where patients would like to receive EoL care, and (iv) preferences relating to participation in a shared decision-making process concerning EoL care.

We excluded studies investigating preferences regarding interventions of limited availability, or interventions whose legal status depends on national legislation, such as euthanasia. We also excluded studies exploring patients’ will to live.

### 2.3. Information Sources and Search

We searched the following electronic sources from inception to November 12th, 2020: MEDLINE, CINAHL, PsycINFO, Social Sciences Citation Index, Social Sciences Citation Index Expanded, PSYNDEX and The Cochrane Library. We followed the recommendations of PRESS Peer Review of Electronic Search Strategies and developed the final search strategy in collaboration with an expert medical science librarian [14]. The electronic search strategy used in the MEDLINE database is provided in Supplementary Table S2. This search strategy was adapted for use in the other databases. To avoid dissemination bias, we did not apply any restrictions to publication date or language.

We also examined the reference lists of included studies, relevant systematic reviews and meta-analyses, and searched for cited references (forward and backward citation tracking) using the Web of Science Collection.

### 2.4. Study Selection

Bibliographic details of all identified references were first imported to Endnote© and then uploaded to COVIDENCE© for title, abstract and full-text screening. Duplicates were removed. Two review authors (A.I.G.-G., J.N.) independently screened the title and abstract of every identified reference to determine which should be assessed further. Before screening, a stepwise calibration exercise was performed on a sample of 30 studies [15], with the aim of achieving at least 80% agreement between reviewers. The full texts of potentially eligible papers were then retrieved and independently assessed for eligibility by two reviewers (A.I.G.-G., J.N.). Any discrepancy was resolved through discussion and, where necessary, consensus involving a third reviewer (C.S.).

### 2.5. Data Collection Process

One review author (A.I.G.-G.) extracted key study and participant characteristics from all studies that fulfilled the inclusion criteria and reported data on outcomes. A second review author (C.S.) cross-checked the data extraction. Any disagreement was resolved by discussion, or, where necessary, with the help of a third author (C.M.).

### 2.6. Data Items

We stratified data according to study type (i.e., qualitative, quantitative, mixed methods observation, interventional) using standard extraction templates in Excel and Access datasheets. Data were extracted and assigned to the following categories: Study reference (i.e., first author, year of publication, country of study origin); Study aim; Study setting; Sample size; Population characteristics (e.g., age, sex, definition of multimorbidity, patient prognosis or illness severity, cancer or non-malignant condition); Preference assessment method (e.g., interview or questionnaire); Context of preference (i.e., hypothetical/real, preference-sensitive situation); Information provided by the authors on the presentation of alternatives (e.g., positive or negative framing [16,17]); Description of phenomenon of interest (EoL care elements that patients were asked about, e.g., resuscitation preference); Results of described phenomenon of interest (e.g., proportion of participants expressing a preference for a specific type of EoL care).

### 2.7. Risk of Bias

One review author (A.I.G.-G.) assessed the risk of bias (RoB) using the Mixed Methods Appraisal Tool (MMAT) [18], which a second reviewer (C.S.) verified. Assessments of the impact of the RoB on further analysis were discussed and, where necessary, involved consultation with a third author (C.M.). Studies were categorized according to study design, whereby the assessment depended on the employed methodology. The tool was adapted to include a column evaluating the existence of a framing effect, which is a cognitive bias that occurs when the way information is presented influences the choices patients make [16,17]. The framing effect was independently assessed by two review authors (A.I.G.-G., M.-S.B.), whereby disagreements were resolved by discussion and, where necessary, involved a third author (C.S.).

### 2.8. Summary Measures

We provided summary statistics for each phenomenon of interest, irrespective of whether a statistical synthesis had been performed. Where possible, we presented the number of participants expressing a preference for a specific type of EoL care in proportion to the total number of participants per study. We reported study-level data both narratively and visually (using graphs) and displayed the results in tabular form.

### 2.9. Synthesis of Results

We conducted a mixed methods systematic review using a convergent integrated approach, whereby we (i) synthesized qualitative data by means of thematic synthesis, (ii) synthesized quantitative data and performed a meta-analysis if applicable, and, in a final step, (iii) synthesized and integrated both (i) and (ii) according to the methodologies described by Sandelowski et al. [19], Pearson et al. [19,20], and the Joanna Briggs Institute [11].

For the qualitative analysis, two reviewers (A.I.G.-G., J.N.) independently analysed the extracted data and provided thematic codes according to the above-mentioned classification of the phenomenon of interest (i.e., type of EoL care preference). Both reviewers discussed coding and identified overarching thematic issues and categories with the help of MAXQDA 18 software [21,22]. Disagreements were resolved by discussion and, where necessary, by involving a third author (C.S.).

For the quantitative analysis, data from the observational and interventional studies were analysed together and included a baseline assessment of EoL care preferences. Meta-analysis of data was considered for studies that had provided comparable and sufficiently homogeneous outcomes. We first assessed heterogeneity qualitatively (in terms of study design, population and the phenomenon of interest), and, assuming the qualitative assessment did not preclude a meta-analysis of the studies, also by means of X^2^ and additional tests. If meta-analysis was impossible, a descriptive analysis was carried out.

For the mixed methods data synthesis (integrated synthesis methodology [19,20]), two reviewers (A.I.G.-G., C.S.) decided which compatible format was the most promising based on the results of (i) and (ii), and involved a third reviewer (C.M.) if consensus could not be reached. The decision depended mainly on the number of qualitative and quantitative studies that were eligible for inclusion [19,20]. Wherever possible, meta-analysis was used to convert qualitative data into a numerical format for quantitative synthesis [19,20,23,24] by using the Chang et al. approach to transform verbal counts into numbers [25].

### 2.10. Additional Analysis

We originally planned to perform sensitivity analyses in case important RoB was detected. However, such analyses were not feasible due to the limited number of studies focusing on the same phenomenon of interest (i.e., type of EoL care preference).

For the same reason, we could not conduct the originally planned subgroup analysis to examine whether EoL care preferences were affected by, for example, age, sex, specific life-sustaining treatment modalities, and specific contexts of the preference assessment (hypothetical or real scenarios).

## 3. Results

After screening 5027 unique references, twenty-two studies were included in the systematic review. We contacted two authors of papers for which we could not find the full text but obtained no response and therefore excluded them due to missing data (Supplementary Figure S1). Supplementary Table S3 presents excluded studies with reasons for exclusion.

### 3.1. Key Characteristics of the Included Studies and Participants

Table 1 and Table 2 show key characteristics of the included 22 studies and 3243 patients (range 12–682). Twenty-one studies used observational designs (three were qualitative, 15 were quantitative and three used mixed methods) and one was a quasi-experimental study.

Of the 15 observational quantitative studies, five [38,39,40,45,47] were conducted in North America, seven [27,32,33,34,35,36,44] in Europe and three [28,31,43] in Asia. The studies were performed between 1992 and 2020 and mostly in an outpatient setting. Of the three qualitative studies, two [41,46] were performed in North America and the other [37] in Europe. All qualitative studies were conducted in an outpatient setting between 2011 and 2018. The number of patients in the quantitative studies ranged from 34 to 682, their mean age was 62 to 83 years and 1175 (42%) of them were female. The number of patients in the qualitative studies ranged from 12 to 146, their mean age was 63 to 70 years and 16 (9%) were female. Of the three studies that used a mixed methods design [26,30,42], one was conducted in Europe in the year 2000 and the other in Europe in 2020. The number of patients ranged from 40 to 82, their mean age was 74 to 84 years and 67 were female [26,30]. The other mixed method study was conducted in North America in 2008, and had 18 patients with a mean age of 74, of which 16 were female [42]. All the mixed methods studies were conducted in an outpatient setting. The quasi-experimental study [29] was conducted in an outpatient setting in China in 2010. It included 121 patients with a mean age of 84 years, of which 84 were female. Nine studies assessed preferences in patients with multimorbidity without any indication of an index disease [28,29,38,39,40,42,45], while the remaining 13 studies assessed patients with index diseases associated with at least one other morbidity (i.e., cancer, cardiovascular disease, chronic heart failure, chronic obstructive pulmonary disease and ESRD). Response rates ranged from 43% to 100% of eligible patients, with a mean response rate of 76% (19).

### 3.2. Risk of Bias within Studies

The results of the RoB assessment are shown in Supplementary Table S4.

All included studies had clear research questions and collected data that addressed the research question. The three qualitative studies [37,41,46] had a low RoB, although authors of one of the studies [46] did not describe the questions patients were asked during the interviews. The only quantitative non-randomized study [29] had low RoB. The quality of the 15 quantitative descriptive studies [27,28,31,32,33,34,35,36,38,39,40,43,44,45,47] varied, with eleven studies [27,31,32,33,34,35,36,38,39,40,44] scoring high because of a high participant non-response bias, three studies [31,40,44] not providing enough information to assess representativeness, and one study [31] not providing enough information to assess whether the employed measurements were appropriate. The three mixed methods studies [26,30,42] also scored low. Overall, five studies [26,29,32,33,36] were considered to have a potential framing effect (cognitive bias caused by the influence of the way information is presented on the choices people make) as selections may have been positively (e.g., survival rates) [26] or negatively (e.g., risk of death) [29,32,33,36] framed by the authors. One study [31] did not provide enough information to assess framing.

All studies were included in the final synthesis, with greater emphasis placed on higher quality studies.

### 3.3. EoL Care Preferences of Older Patients with Multimorbidity

Among the included studies, 19 [26,27,28,29,31,32,33,34,36,37,38,39,40,42,43,44,45,46,47] reported data on willingness to receive LSTs; 14 reported data on specific LSTs such as cardiopulmonary resuscitation (CPR) [26,31,32,33,34,36,38,40,42,47], mechanical ventilation (MV) [31,32,33,34,36,37,38,42,45] via endotracheal intubation (ETI) [27] or non-invasively (NIV) [27], use of feeding tubes [38,42,43], blood transfusion [38], dialysis [42,47] and implantable cardioverter defibrillator (ICD) withdrawal [46]. Three studies [27,30,40] reported on patients’ willingness to opt for palliation of symptoms, six studies [30,33,35,36,38,45] on the patients’ preferred place of care, and eight [26,28,31,37,41,42,44,46] on the wish to be involved in shared decision-making concerning EoL care. Sixteen studies [26,27,28,30,31,33,36,37,38,40,41,42,44,45,46,47] addressed more than one type of EoL care.

#### 3.3.1. Willingness to Receive LSTs

Five studies explored willingness to receive LSTs without specifying the type of intervention [28,45] or by combining two or more LSTs [29,39,44] (Table 3).

**Figure 1:**
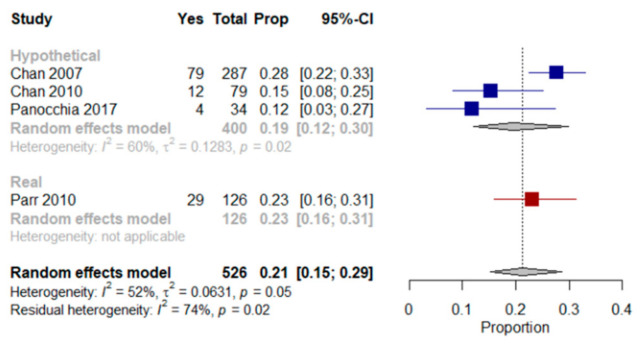
Forest plot comparing the willingness to receive life-sustaining treatments: Shows results (%) from studies where the context was a hypothetical scenario, a real scenario or regardless of the scenario patients were presented with when end-of-life care preferences were assessed.

We were able to perform a meta-analysis based on four [28,29,44,45] of the five studies mentioned above. The results showed that a mean proportion of 21% (95% CI, 15–29%, I^2^ = 52%) of older patients with multimorbidity were willing to receive LSTs regardless of the scenario authors presented to them (real and/or hypothetical). Results from a meta-analysis based on the studies in which patients were presented with only hypothetical scenarios revealed that a mean proportion of 19% (95% CI, 12–30%, I^2^ = 60%) of patients were willing to receive LSTs (Figure 1).

Parr et al. [45] assessed EoL treatment preferences in older patients with multimorbidity, advanced cancer and a life expectancy of less than six months using a real scenario. Twenty-three percent of those patients preferred a course of LST treatment aimed at extending life to the greatest possible extent, even if it meant more pain and discomfort.

Chan et al. studies [28,29] assessed preferences for LST in older Chinese patients with multimorbidity that lived in long-term care homes. They were presented with a hypothetical scenario and asked, “if you were severely ill with a life-threatening condition, and LSTs could help you extend your life but not restore your health, would you want to receive them?”. Between 12% and 42% of patients were undecided as to whether LSTs should be used in a life-threatening situation. In Panocchia et al. [44], 12% of patients with end-stage renal disease (ESRD) chose to undergo MV or PEG in at least one of the proposed hypothetical scenarios (i.e., persistent vegetative status, terminal illness and dementia); the authors did not mention the number of patients who refused or were not certain how to respond. Menon et al. [39] used a modified Treatment Preferences Questionnaire [38] to assess the desire for LSTs in older patients with multimorbidity that had been admitted to an acute medical unit. Patients were asked to state their preference in the current situation (i.e., during ongoing hospitalization) and after imagining one of six possible illness scenarios (i.e., stroke, diabetes, stomach cancer, Alzheimer’s disease plus kidney failure, arthritis plus pneumonia and brain cancer). The Treatment Preference Questionnaire scores for patients with depression but low hopelessness, no depression but high hopelessness and depression plus high hopelessness were compared. A statistically significant association was not found for depression but was observed between hopelessness and the desire for fewer LSTs, which remained statistically significant after adjusting for race, religiousness and education. Statistically significant associations were found between high hopelessness and the desire for fewer LSTs in the dementia-associated-with-kidney-failure and arthritis-with-pneumonia scenarios.

**Figure 2:**
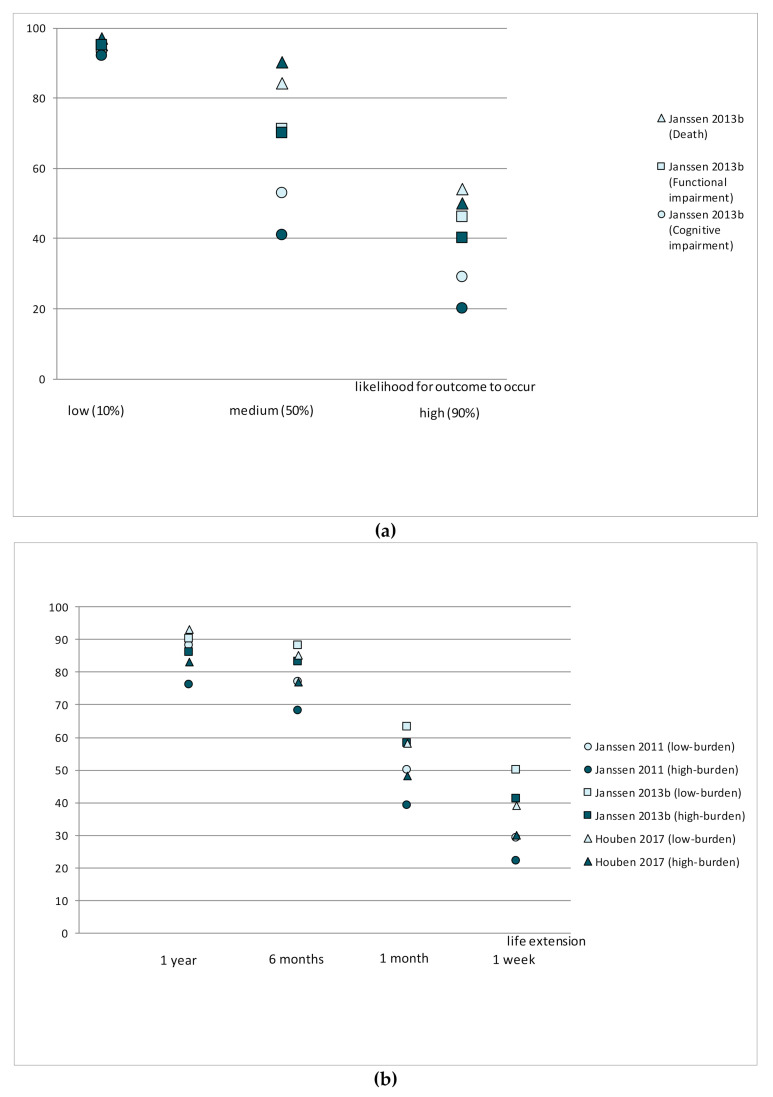
Willingness to receive life-sustaining treatments in different scenarios: (**a**) Compares preferences (%) from two studies assessing the willingness to receive LSTs depending on the risk of death, cognitive or functional impairment (%); (**b**) Compares preferences (%) from three studies assessing the willingness to receive LSTs depending on life extension and treatment burden.

Three studies [32,33,36] used the Willingness to Accept Life-Sustaining Treatment (WALT) instrument to identify LST preferences with respect to treatment burden, treatment outcomes, and likelihood of those outcomes [48] (Figure 2). The WALT questionnaire consists of six scenarios in which patients weigh treatment burden against treatment outcome based on the likelihood of different health states and extension of life following treatment [48]. The proportion of patients choosing LSTs decreased in line with an increase in the likelihood that the outcome of the treatment would be death or functional and/or cognitive impairment. Similarly, the proportion of patients choosing a treatment increased in line with the life extension that was expected to result from it.

In a longitudinal study, Janssen et al. [34] investigated the stability of CPR and MV preferences in outpatients with stable but advanced multimorbidity over one year. CPR and/or MV preferences changed at least once in 38% of patients during the follow-up period. Fourteen percent of patients that preferred CPR and/or MV at baseline reported less willingness after one year, and 6% reported swings in their preferences over the period. Eleven percent of patients that refused CPR and/or MV reported greater willingness after one year, and 5% reported a change in their preferences at some point during the study period. In Chan et al.’s quasi-experimental study [29], treatment preference stability was significantly higher in the group that participated in an ACP program (i.e., intervention) (*p* ≤ 0.001).

##### Willingness to Receive CPR

Ten studies [26,31,32,33,34,36,38,40,42,47] reported on the willingness of older patients with multimorbidity to receive CPR (Table 3). With the exception of Fuseya et al. [31], studies assessing patients’ preferences for CPR in their current state of health, showed that 73 to 90% of included patients expressed their willingness to receive CPR.

**Figure 3:**
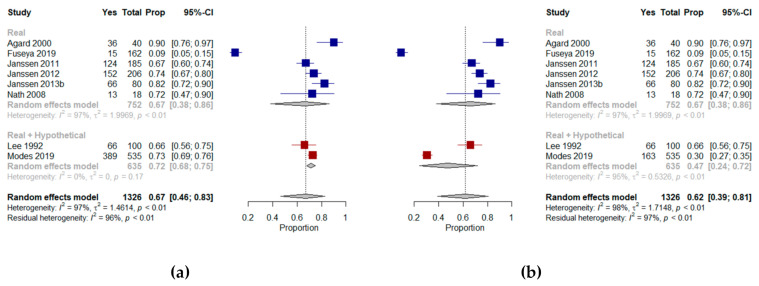
Forest plots comparing willingness to receive cardiopulmonary resuscitation: (**a**) Shows results (%) from studies where the context was a real scenario or both scenarios and includes the results from Modes et al. assessing patients’ preferences in their current state of health; (**b**) Shows results (%) from studies where the context was a real scenario or both scenarios and includes the results from Modes et al. assessing patients’ preference in a hypothetical state of dependency.

We were able to include eight of the 10 studies in a meta-analysis [26,31,33,34,36,38,40,42], which showed that a mean proportion of 67% (95% CI, 46–83%, I^2^ = 97%) of older patients with multimorbidity were willing to receive CPR in their current state of health. A meta-analysis of two studies [38,39] that included hypothetical scenarios of a deterioration in health showed that a mean proportion of 47% (95% CI, 24–72%, I^2^ = 95%) of older patients with multimorbidity were willing to receive CPR (Figure 3).

Agard et al. [26] explored willingness to undergo CPR by presenting patients with chronic heart failure with different chances of survival following a resuscitation attempt; the majority (90%) opted for CPR, regardless of their chances of survival. Tamura et al. [47] reviewed the medical records of older patients with ESRD and found that 17% of those that had discussed EoL with a health professional had do-not-resuscitate orders. Janssen et al. explored preferences for CPR in Dutch outpatients with severe multimorbidity in three different studies [33,34,36]. Most reported that they would prefer to receive CPR, with only a few patients (2–3%) unable to express a preference. Changes in generic health status, mobility, symptoms of anxiety and depression, and marital status over time were associated with changes in CPR preferences [34]. In a study of African American patients with multimorbidity, Nath et al. [42] found that almost two-thirds wanted CPR if indicated. A comparison between documented (according to advance directive data) and stated preferences for CPR showed that although 16 of the 18 participants had a documented advance directive requesting CPR should they become ill, three of them reported that they did not want CPR under their current circumstances.

Lee et al. [38] determined the effect of depression on EoL preferences for CPR in older veterans with multimorbidity. Thirty-four percent of patients refused CPR in their present state of health and under each of the four scenarios that were presented (i.e., pneumonia in a wheelchair-bound older person requiring assistance with personal care, a massive stroke with a high risk of death and low likelihood of functional recovery, iatrogenic renal failure in a functionally independent person with a good chance of complete recovery and a life-threatening gastric hemorrhage in a person with advanced cancer experiencing no pain or cognitive impairment and whose life expectancy could be as long as six months). No link to depression was found in either acceptors or refusers. However, authors reported that refusers of CPR were characterized by significantly lower quality of life than acceptors. Modes et al. [40] investigated the CPR preferences of older patients with multimorbidity facing explicit hypothetical trade-offs. Patients were offered the choice between life extension and the relief of discomfort in their current state of health or in a state of dependency. Patients that prioritized extending life were most likely to prefer CPR, with 93% preferring CPR in current health, and 67% preferring CPR when dependent on others, compared with 69% and 21% respectively in patients prioritizing relief of discomfort, and 78% and 33% respectively in patients that were unsure whether they preferred life extension or relief of discomfort.

Fuseya et al. [31] evaluated preferences for CPR in Japanese patients with multimorbidity with severe COPD. Nine percent of the patients wanted to receive CPR, 15% indicated they did not want to receive CPR and almost half the patients indicated they could not yet make a decision on possible invasive treatment in the future.

##### Willingness to Receive Mechanical Ventilation (MV)

Nine studies [27,31,33,34,36,37,38,42,45] assessed preferences for MV in older patients with multimorbidity (Table 3). In the majority of the studies [33,34,36,38,42], patients expressed their willingness to receive indicated MV in their current state of health (between 56–81% of patients).

**Figure 4:**
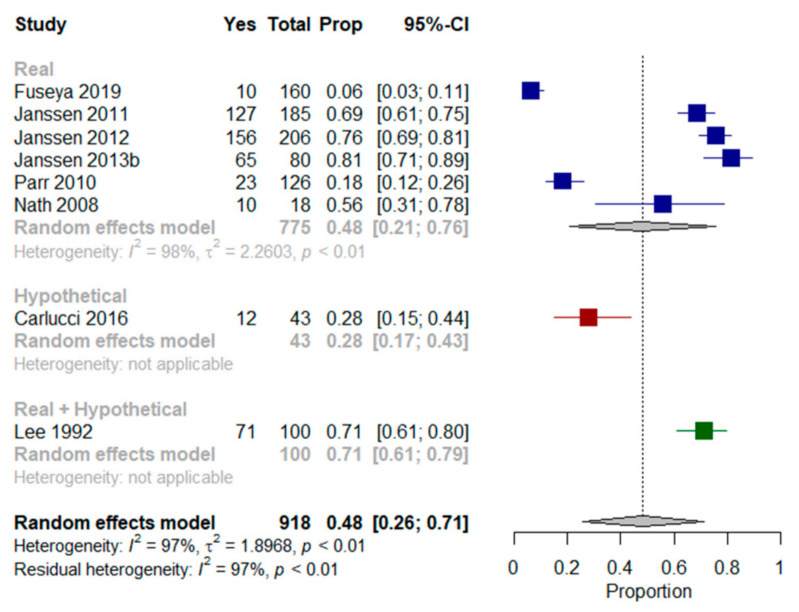
Forest plots comparing willingness to receive mechanical ventilation: Shows results (%) from studies where the context was a real scenario, a hypothetical scenario or both scenarios.

We performed a meta-analysis on six of the nine studies [31,33,34,36,42,45] assessing the preferences of patients in their current state of health. The results showed that a mean proportion of 48% (95% CI, 21–76%, I^2^ = 98%) of older patients with multimorbidity were willing to receive MV. When findings from Carlucci et al. [27] and Lee et al. [38] (who presented patients with both real and hypothetical scenarios) were included in the meta-analysis, the proportion of patients willing to receive MV remained at 48% (95% CI, 26–71%, I^2^ = 97%) (Figure 4).

Janssen et al. studies [33,34,36] explored preferences for MV. Most included patients with multimorbidity indicated they would prefer to receive it. As for CPR, changes in generic health status, mobility, symptoms of anxiety and depression, and marital status over time, were associated with changes in CPR preferences [34]. Nath et al. [42] found that half the patients wanted MV if necessary. Lee et al. [38] found that 29% of patients refused MV in their present state and under every presented scenario, with no association with depression identified in either acceptors or refusers. However, the authors reported that refusers of MV had a significantly lower quality of life than acceptors. Carlucci et al. [27] used a scenario-based decision aid to elicit preferences in patients with multimorbidity and severe COPD. They were offered the choice between receiving MV via ETI, or “ceiling” NIV, and palliation of symptoms with oxygen and morphine. Thirty percent of patients indicated a preference for MV via ETI over the other two options. In Jerpseth et al. [37], patients with severe COPD said they would appreciate having the opportunity to use a mask as a “life buoy”, a symbol of hope and survival—even when there was no chance of complete recovery. All patients that had received MV subsequent to NIV treatments in the previous year (50% of included patients) said they would not undergo such treatment again.

In Fuseya et al. [31], six percent of patients with severe COPD wanted to receive MV, and 18% indicated they did not want to receive MV. With regard to CPR, more than half indicated they had not made a decision on whether to receive any invasive life-extending treatment in the future. In Parr et al. [45], 18% of older patients with very advanced cancer stated a preference for treatment that focused on the use of ventilators to extend life, even if it meant more pain and discomfort.

##### Willingness to Receive Other Life-Sustaining Treatments (LSTs)

Four studies [38,42,43,46] assessed willingness to receive other LSTs (Table 3). Lee et al. [38] found that in their present state and under every scenario that was presented, intravenous therapy, blood transfusions and nasogastric tubes were accepted by 93%, 89% and 52% of patients with multimorbidity respectively. Nath et al. [42] investigated the preferences of frail patients with multimorbidity regarding the insertion of a feeding tube. Although most participants indicated a preference, a small group reported being “unsure”. Ni et al. [43] reported that more than half the participating Chinese nursing home residents expressed a preference for the possible use of a feeding tube in case of severe cognitive impairment or even a life-threatening condition in which tube feeding could only help to sustain life but not result in a return to current health. The reasons for the willingness to accept the use of a feeding tube were “I should try when there is a chance” (84%) and “desire to live longer” (10%); reasons given for rejecting a feeding tube included “lived life long enough” (39%) and “doubts about the effectiveness of LST” (30%). Strachan et al. [46] assessed willingness to remove an ICD in older patients with multimorbidity and severe cardiovascular disease. Twenty percent of the included patients decided to have the ICD removed. The aim of those who accepted was to prevent sudden cardiovascular death.

##### Willingness to Withdraw from Dialysis

Two studies [44,47] explored the willingness of older patients with multimorbidity and ESRD in outpatient settings to withdraw from hemodialysis (Table 4). Tamura et al. [47] characterized patient preferences for dialysis withdrawal. Two percent of participants would probably or definitely withdraw in their current state of health, 15% in the event of a moderate stroke, 33% in the event of dementia, 32% in the event of terminal cancer and 59% in the event of a coma. Panocchia et al. [44] evaluated preferences regarding a continuation of dialysis treatment. More than half the patients (59%) would continue dialysis in at least one of the proposed scenarios (i.e., persistent vegetative status, terminal illness owing to advanced cancer, heart or liver failure, and dementia with severe cognitive impairment).

#### 3.3.2. Willingness to Opt for Palliation of Symptoms

Two studies assessed willingness to opt for palliation of symptoms [27,40] (Table 3 and Table 4). In Carlucci et al. [27], 42% and 28% of older patients with very severe COPD preferred NIV and MV respectively, and 30% preferred the palliation of symptoms with oxygen and morphine. In a study by Etkind et al. [30], which included frail, older patients with multimorbidity that had recently had an acute illness, life extension was considered important by 43% of participants and was stable in 61% of cases after a follow-up period of six months. Other preferences, such as improving quality of life, which remained stable in 76% of participants during the study period, were more highly rated. According to the authors, preference stability was supported by the presence of family support, both positive and negative care experiences, the concordance of preferences with underlying values, a slow recovery from illness, and when preferences were linked to long-term goals. In Modes et al. [40], 60% of 535 older patients with multimorbidity and a median survival of approximately two years (18% with cancer), prioritized relief of discomfort overextending life, while 17% preferred life extension to relief of discomfort, and 23% were unsure.

#### 3.3.3. The Place of EoL Care

Five studies [33,35,36,38,45] assessed patients’ preferences regarding the place they would like to receive EoL care.

**Figure 5:**
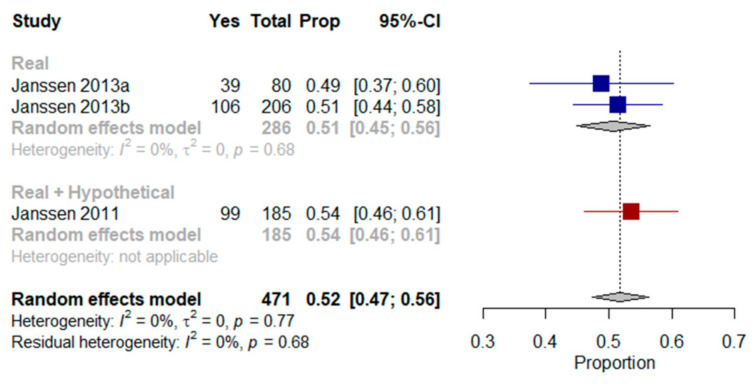
Forest plot comparing the preference for home as a place of end-of-life care: Shows results (%) from studies where the context was a real scenario or both scenarios.

A meta-analysis of three studies was performed [33,35,36]. When asked in their current state of health or using hypothetical scenarios, the results showed that a mean of 52% (95% CI, 47–56%, I^2^ = 0%) and 51% (95% CI, 45–56%, I^2^ = 0%) of older patients with multimorbidity respectively would prefer to die at home (Figure 5).

**Figure 6:**
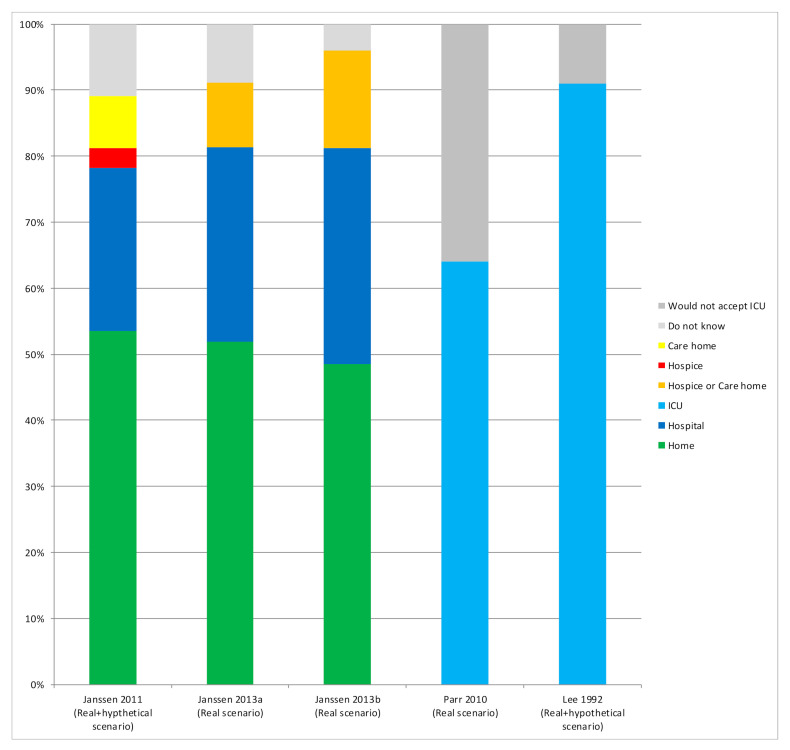
Preferences regarding place of end-of-life care: Shows results (%) from studies that assessed preferences regarding the place of end-of-life care.Two studies [38,45] assessed patients’ preferences for ICUs as a place to receive EoL care (Figure 6). Thirty-six percent of patients with advanced cancer and a life expectancy of less than six months were against dying in an ICU [45], compared with 91% of older adults with multimorbidity but a life expectancy over six months, who considered dying in an ICU to be a ‘bad death’ [38].

Three studies [33,35,36] reported the preferences of older patients with severe multimorbidity in specialized outpatient settings (Figure 6). About half the patients (49–54%) would prefer to die at home, whereas 25–33% of patients would prefer to die in a hospital, and 3–15% in a hospice or a care home. Between 4% and 11% of the patients did not know where they would prefer to die. Janssen et al. [35] also examined the one-year stability of preferences concerning place of death in older patients with multimorbidity and advanced diseases. At one-year follow-up, 61% of patients had changed their preference at least once.

#### 3.3.4. Preferences Regarding Participation in an EoL Shared Decision-Making Process

Seven studies [26,28,31,37,41,42,44] investigated preferences regarding who should be involved or responsible for EoL shared decision-making (Table 5).

In Agard et al. [26], the majority of patients with chronic heart failure (70%) said they would like their physicians to bring up CPR and welcomed the opportunity to discuss the issue: “I want to influence the decision as long as I am in full possession of my faculties”. Chan et al. [28] performed a study in Chinese residents of long term care homes on who should be involved in EoL treatment decisions. The physician was selected by 37% of participants, with “depends on the situation” being the second most popular choice (31%). In Panocchia et al. [44], 91% of patients with ESRD would prefer family members to be involved in the decision-making process.

Chan et al. [28] reported on whose decision Chinese residents regarded as the most crucial in making treatment decisions. The majority of participants (50%) chose the physician as the most important, followed by “depends on the situation” (29%). In Fuseya et al. [31], almost half the patients with severe COPD, when asked who should decide about CPR and MV, selected the option “refer to the physician” (44% and 43% respectively) or “refer to family” (14% and 14% respectively). In Jerpseth et al. [37], participants with severe COPD differed as to how much information and how many conversations they wanted to have on difficult topics. Two (17%) further participants thought “not knowing” was the best option. Two (17%) participants took it for granted that physicians would decide on the course of treatment. The other 10 wanted to play a part in the decision-making process. They all said their care and treatment options depended on their physician’s opinion of their conditions and saw no need to be involved themselves. In Naik et al. [41], 70% of participants with multimorbidity expressed their opinions on the extent to which they and their family and health professionals should participate in their EoL decisions. Many participants saw a need for openness regarding their condition and treatment. For some participants, telling the truth and honesty about one’s prognosis were important aspects of openness. Many respondents also mentioned the importance of collaborative decision-making and entrusted significant others with the responsibility to decide. Other participants discussed asking their doctors to help them in the decision-making process. In Nath et al. [42], participants with multimorbidity were asked about preferences regarding consultations with family members and health professionals when making important EoL decisions. Unless incapacitated or otherwise unable to do so, most preferred to make such decisions independently. When asked, “Do you ever let anyone make decisions for you?”, some answered: “My family don’t make medical decisions unless I’m unconscious and I am under a doctor’s care and I ask him questions about certain things and so I make the decisions for me”. Nevertheless, when asked, 89% of patients had already designated a proxy decision maker in their advance directives. In Panocchia et al. [44], 73% of patients with ESRD would delegate treatment decisions to family members or friends if they were incapable of deciding themselves.

In Strachan et al. [46] several participants said it would be better to discuss the function of ICDs in relation to EoL care while they were still cognitively intact. Those who had already discussed the topic—what they referred to as “the talk”—with their health professionals said it was best to do so when they were already engaged in discussing the pros and cons of the ICD. Several participants said it was an important discussion to have and that they would bring it up next time they went to the ICD clinic.

## 4. Discussion

Patients with multimorbidity often have to make numerous and conflicting decisions and choices, which makes eliciting their preferences rather challenging. Our study provides the first systematic review of EoL care preferences in older patients with multimorbidity. Multimorbidity affects the majority of older adults [49], so therefore the results from this systematic review may apply to a wider older population.

### 4.1. Summary of Evidence

In four studies [28,29,39,44,45], any LST option was preferred by 21% of older patients with multimorbidity. However, between 12–42% of them did not prefer or had not established a preference for any specific LST options because the alternatives had not been explicitly discussed. In another three studies [32,33,36], the proportion of patients choosing LST decreased as death, functional and cognitive decline became more likely, and increased according to potential life extension following treatment.

Overall, the majority of older patients with multimorbidity were willing to receive specific LSTs such as CPR, MV or to continue dialysis when current preferences were assessed during their existing state of health (real scenarios) [26,27,34,36,38,40,42,46,47,50] and decreased under certain hypothetical circumstances (i.e., terminal illness or state of dependency) [40,44,47]. In a systematic review, De Decker et al. [51] showed that do-not-resuscitate orders are positively associated with multimorbidity, particularly in patients with cognitive impairment, cancer and stroke.

Only one study [34] addressed changes in LST preferences after one year. This study concluded that more than one third of patients had changed their preferences regarding willingness to receive CPR or MV. One quasi-experimental study [29] showed significantly higher treatment stability in a group that had participated in an ACP program. According to Auriemma et al. [52] systematic review on EoL preferences, preference stability was generally greater among adult inpatients (80%) and seriously ill adult outpatients (75%) than among older adults with multiple serious diseases (63%). Patients who had engaged in ACP had greater preference stability, and preferences to forgo therapies were generally more stable than preferences to receive therapies. Emanuel et al. [53] systematic review that assessed stability of advance treatment decisions of patients and the general public, including illness scenarios, noted similar levels of stability (93%) among seriously ill adult outpatients and members of the general public, especially for the ones who had discussions with their physicians. It is therefore important to develop systems that enable patients with multimorbidity to regularly discuss EoL care preferences with health care professionals and to repeat assessments of their holistic needs and to review their ACPs where necessary, for example at key transition points, such as when the patient’s health status or treatment goals change.

A key principle of a ‘good death’ is the option to decide where death should occur. In ideal circumstances, half the patients preferred to die at home [33,35,36]. However, in the only longitudinal study we found, preferences regarding the place of death had changed at one-year follow-up [35]. Gomes et al. [54] confirmed our findings that most patients (adult patients facing a real or hypothetical scenario of being in the advanced or severe stages of a progressive disease) prefer to die at home, and that 60% of patients changed their preference as their illness progressed. However, Hoare et al. [55] pointed out that the exact proportion of patients preferring to die at home or anywhere else is unknown, as reported preferences often exclude the views of those with no preference (4–11% according to our review), and of those that were not asked/considered (or did not respond—the non-response rate was 25–46% according to our review). There is therefore an urgent need for studies that examine changes in the place older patients with multimorbidity would prefer to receive EoL care.

Older patients with multimorbidity prefer not to delegate decisions concerning their own EoL care unless incapacitated [26,37,41,42,44]. However, they are inclined to involve their physicians and family members in the decision-making process [26,29,31,37,41,42,44]. Health professionals should support and enable patients approaching EoL by preparing to review and anticipate patients’ information needs and preferences in advance and as circumstances change. This process should involve assessing the appropriate amount and type of information that patients would like to receive. If desired by the patient, caregivers and other individuals important to them should be included in the discussions.

### 4.2. Limitations

Several limitations of the review should be considered when interpreting the results. First, the review included studies with varying features and populations. Such methodological differences (e.g., data collection methods, quality of the studies) and clinical differences (e.g., type of conditions, life expectancy/severity of the patient population) may partly explain variations in our findings.

Second, the relatively small samples sizes in some of the evaluations of phenomena of interest may have limited the generalizability of our results (e.g., preferred place of death).

Third, the individual studies assessed a convenience sample of patients without examining the differences between participants and non-participants, and non-response rates were high. It is unknown whether and to what extent this influenced the results.

Fourth, EoL care preferences, like preferences in general, are influenced by how and under what circumstances the information is presented to patients. Some patients provided their preferences in ideal circumstances, whereas others described them while in a difficult situation. As circumstances impact preferences for a specific EoL intervention, they may have influenced our results. To adjust for this effect, we separated studies according to context for the meta-analysis, i.e., whether patients were presented with hypothetical scenarios, or responding in the light of their current situation. The setting (i.e., whether patients were hospitalized or in an outpatient setting), the patient’s conditions (e.g., cancer or non-malignant), patient prognosis, illness severity, information provided by authors when presenting alternatives, as well as other factors we did not address in this review (e.g., marital status, ethnicity, religiosity, functional status, quality of life, symptoms of anxiety, depression with or without hopelessness, previous experience of the intervention…) may influence patients’ preferences and should therefore be considered when interpreting the results. In order to gain a better understanding of patients’ EoL care preferences, further research should clarify how preferences should be elicited and under what circumstances.

Fifth, all but two of the included studies asked patients about their preferences on only one occasion, although participants may well reconsider their EoL care preferences in response to changes in, for example, their health status or living situation.

Finally, caregivers, family members, and other people that are important to older patients with multimorbidity and that are likely to be asked for their opinion in EoL situations, were not included in this review. Health care professionals’ views were not taken into account either. Differences may exist between a patient’s preferences and the perceptions of caregivers and health professionals, who are not always aware of the patient’s preferences but play a very important role in the care of terminally ill patients with multimorbidity.

## 5. Conclusions

This review assesses EoL care preferences of older patients with multimorbidity. Such preferences need to be understood in the context of patients’ knowledge and expectations of supportive (i.e., available LSTs) and palliative care. Patients should therefore receive information on treatment burden and expected outcomes, including the likelihood of an adverse treatment outcome, when discussing EoL care preferences. This will allow patients to make carefully considered and informed decisions about LSTs and alternative conservative care options.

By inquiring about the EoL care preferences of older patients with multimorbidity, health professionals can help ensure that the care provided to this patient population is concordant with the care that patients desire.

## Figures and Tables

**Table 1:** Key characteristics of the included studies.

Author, Year (Reference)	Country	Setting	Design	Scenario	Data Collection Methods	Response (%)	Participants, *n*	Female Sex, %	Condition	EoL Care Preferences Under Review
Agard, 2000 [26]	Sweden	Outpatient (specialized)	Observational, mixed methods	R	Interview Semi-structured interview	87	40	38	CHF (NYHA II-IV) + CM	CPR, SDM
Carlucci, 2016 [27]	Italy	Inpatient	Observational, quantitative cross-sectional	H	Semi-structured interview	47	43	16	COPD (very severe) + CM	ETI, NIV, palliation of symptoms
Chan, 2007 [28]	China	Outpatient (nursing home)	Observational, quantitative cross-sectional	H	Questionnaire	86	287	78	MM (frail and non-frail)	LST, SDM
Chan, 2010 [29] *	China	Outpatient (nursing home)	Interventional	H	Questionnaire	93	121 (IG 59; CG 62)	69	MM (frail)	LST
Etkind, 2020 [30]	United Kingdom	Outpatient (specialized)	Observational, mixed methods	R	Questionnaire	43	82	63	MM (frail)	Quantity versus quality of life
Fuseya, 2019 [31]	Japan	Outpatient (specialized)	Observational, quantitative cross-sectional	R	Questionnaire	71	160	7	COPD (severe) + CM	CPR, MV, SDM
Houben, 2016 [32] ^#^	The Netherlands	Outpatient (specialized)	Observational, quantitative longitudinal	R & H	Questionnaire (WALT) (47)	78	206	36	CHF (NYHA III-IV), COPD (GOLD III-IV), or ESRD + CM	CPR, MV
Janssen, 2011 [33]	The Netherlands	Outpatient (specialized)	Observational, quantitative cross-sectional	R & H	Semi-structured interview Questionnaire (WALT) (47)	54	185	36	CHF (NYHA III-IV), COPD (GOLD III-IV) + CM	CPR, MV, place for EoL care
Janssen, 2012 [34] ^#^	The Netherlands	Outpatient (specialized)	Observational, quantitative longitudinal	R	Semi-structured interview	59	206	36	CHF (NYHA III-IV), COPD (GOLD III-IV), or ESRD + CM	CPR, MV
Janssen, 2013a [35] ^#^	The Netherlands	Outpatient (specialized)	Observational, quantitative longitudinal	R	Semi-structured interview	59	206	36	CHF (NYHA III-IV), COPD (GOLD III-IV), or ESRD + CM	Place for EoL care
Janssen, 2013b [36]	The Netherlands	Outpatient (specialized)	Observational, quantitative cross-sectional	R & H	Questionnaire (WALT) (47)	73	80	40	ESRD + CM	CPR, MV, place for EoL care
Jerpseth, 2018 [37]	Norway	Outpatient (specialized)	Observational, qualitative cross-sectional	R	Interview	100	12	58	COPD (GOLD III-IV) + CM	MV, NIV, SDM
Lee, 1992 [38]	USA	Inpatient	Observational, quantitative cross-sectional	R & H	Questionnaire	71	100 (50 depressed & 50 non-depressed)	5	MM (life expectancy over 6 months)	Blood transfusion, CPR, feeding tube, intravenous fluids with medication, MV, place for EoL care
Menon, 2000 [39]	USA	Inpatient	Observational, quantitative cross-sectional	R & H	Questionnaire (Treatment Preferences Questionnaire) [38]	71	295 (236 no depression, 59 major depression)	0	MM	LST (blood transfusion or CPR or feeding tube or intravenous fluids with medication or MV)
Modes, 2019 [40]	USA	Outpatient (specialized)	Observational, quantitative cross-sectional	R & H	Questionnaire	58	535	47	MM (Median survival approx. 2 years, 18% cancer)	CPR, palliation of symptoms
Naik, 2016 [41]	USA	Outpatient (specialized)	Observational, qualitative cross-sectional	H	Interview	86	146	2	Advanced cancer + CM	SDM
Nath, 2008 [42]	USA	Outpatient (community)	Observational, mixed methods	R	Semi-structured interview & Chart review	90	18	89	MM (frail)	CPR, feeding tube, MV, SDM
Ni, 2020 [43]	China	Outpatient (nursing home)	Observational, quantitative cross-sectional	H	Questionnaire	100	682	61	MM (frail)	Feeding tube
Panocchia, 2017 [44]	Italy	Outpatient (specialized)	Observational, quantitative cross-sectional	H	Questionnaire	62	34	44	ESRD + CM	Dialysis withdrawal, LST (MV or PEG), SDM
Parr, 2010 [45]	USA	Outpatient (specialized)	Observational, quantitative cross-sectional	R	Semi-structured interview	100	126 °	41	MM (life expectancy less than 6 months)	LST, MV, place for EoL care
Strachan, 2011 [46]	Canada	Outpatient (specialized)	Observational, qualitative cross-sectional	R	Interview	100	30	20	CVD + CM	ICD Quantity versus quality of life, SDM
Tamura, 2010 [47]	USA	Outpatient (specialized)	Observational, quantitative cross-sectional	R & H	Questionnaire (Dialysis Living Will) & Chart review	98	61	26	ESRD + CM	CPR, Dialysis withdrawal

CG = Control Group; CHF = Chronic Heart Failure; CM = Comorbidities; COPD = Chronic Obstructive Pulmonary Disease; CPR = Cardiopulmonary Resuscitation; CVD = Cardiovascular Disease; ETI = Endotracheal intubation; ESRD = End Stage Renal Disease; H = Hypothetical; ICD = Implantable Cardioverter Defibrillator; IG = Intervention Group; LST = Life-Sustaining Treatment; MM = Multimorbidity; MV = Mechanical Ventilation; NIV = ‘ceiling’ non-invasive ventilation; *n* = number; nr = not reported; PEG = Percutaneous Endoscopic Gastrostomy; R = Real; SDM = Shared Decision-Making; WALT = Willingness to Accept Life-Sustaining Treatments. * The intervention was a Let Me Talk Advance Care Planning Program (storytelling approach). ^#^ Same population. ° Only “Older aged”, defined as 65 years or older, are included.

**Table 2:** Descriptive summary of included studies.

Variable	Total—*n* (%)
**Study characteristics**	
Geographical location	
- North America	8 (36%)
- Europe	10 (46%)
- Asia	4 (18%)
Setting	
- Outpatient (specialized)	15 (68%)
- Outpatient (nursing home)	3 (14%)
- Outpatient (community)	1 (4%)
- Inpatient	3 (14%)
Design	
- Quantitative	16 (73%)
○ Cross-sectional	12 (41%)
○ Longitudinal	3 (14%)
○ Interventional	1 (4%)
- Qualitative	3 (14%)
○ Cross-sectional	3 (14%)
- Mixed methods	3 (14%)
- Observational	21 (96%)
- Interventional	1 (4%)
Data collection methods *	
- Interview	4 (18%)
- Semi-structured interview	7 (32%)
- Questionnaire	13 (59%)
- Chart review	2 (9%)
Context	
- Real scenario	9 (41%)
- Hypothetical scenarios	6 (27%)
- Both	7 (32%)
Sample size—Total (range) †	3.243 (12–682)
- Observational	
○ Quantitative †	2.794 (34–682)
○ Qualitative	188 (12–146)
○ Mixed methods	140 (18–82)
- Interventional	121
**Patients’ characteristics**	
Type of condition *	
- Studies describing patients with multimorbidity	9 (41%)
- Studies describing patients with a chronic condition associated with multimorbidity	
○ Cardiovascular disease	1 (4%)
○ Chronic heart failure	5 (23%)
○ Chronic obstructive pulmonary disease	7 (32%)
○ End-stage renal disease	6 (27%)
○ Cancer	2 (9%)
Age (range)	62–84
Sex (% female)	1.358 (42%)

* Studies may be included in more than one category. † Studies including the same participants were counted only once [32,34,35].

**Table 3:** Willingness to receive LSTs.

Author, Year (Reference)	EoL Care Measure	Scenario	Type of Condition	Participants, *n*	Yes, *n* (%)	No, *n* (%)	Do Not Know/No Opinion, *n* (%)
Agard, 2000 [26]	CPR	R	CHF (NYHA II-IV) + CM	40	36 (90)	2 (5)	2 (5)
Carlucci, 2016 [27]	MV	H	COPD (very severe) + CM	43	12 (28)	nr	nr
	NIV				18 (42)		
	Only oxygen and drugs				13 (30)		
Chan, 2007 [28]	LST (non-specified)	H	MM	287	79 (28)	88 (31)	120 (42)
Chan, 2010 [29] *	LST (CPR/MV)	H	MM	79	12 (12)	55 (55)	12 (12)
Fuseya, 2019 [31]	CPR	R	COPD (severe) + CM	160	15 (9)	24 (15)	121 (76) ^¥^
	MV				10 (6)	29 (18)	121 (76)
Janssen, 2011 [33]	CPR	R	CHF (NYHA III-IV), COPD (GOLD III-IV) + CM	185	124 (67)	nr	5 (3)
	MV				127 (69)		7 (4)
Janssen, 2012 [34] (same population as Janssen, 2013a [35] and Houben, 2017 [32]) ^ž^	CPR	R	CHF (NYHA III-IV), COPD (GOLD III-IV), or ESRD + CM	206	52 (74)	51 (25)	3 (2)
	MV				156 (76)	44 (21)	6 (3)
Janssen, 2013b [36]	CPR	R	ESRD + CM	80	66 (83)	14 (18)	0
	MV				65 (81)	13 (16)	
Jerpseth et al. [41]	MV	R	COPD (GOLD III-IV) + CM	12	nr	6 (50)	nr
Lee, 1992 ^§^ [38]	CPR	R & H	MM (life expectancy over 6 months)	100	66 (66)	34 (34)	0
	MV				71 (71)	29 (29)	0
	IV				93 (93)	7 (7)	0
	BT				89 (89)	11 (11)	0
	NG				52 (52)	48 (48)	0
Modes, 2019 [40]	CPR	R	MM (median survival approx. 2 years, 18% cancer)	535	389 (73)	129 (24)	0
		H			163 (31)	355 (66)	
Nath, 2008 [42]	CPR	R	MM (frail)	18	13 (72)	2 (11)	0
	MV				10 (56)	8 (44)	0
	Feeding tube				9 (50)	4 (22)	5 (28)
Ni, 2020 [43]	Feeding tube	H	MM (frail)	682	372 (55)	310 (45)	0
Panocchia, 2017 [44]	LST (MV/PEG)	H	ESRD + CM	34	4 (12)	nr	nr
Parr, 2010 [45]	LST (non-specified)	R	Advanced cancer + CM (life expectancy < 6 months)	126 ^#^	29 (23)	97 (77)	0
	MV				23 (18)	103 (82)	
Strachan, 2011 [46]	ICD	R	CVD + CM	30	24 (80) accepted (in the past)	6 (20) declined (in the past)	0
Tamura, 2010 [47] ^£^	CPR	R	ESRD + CM	61	nr	6 (10)	nr

BT = Blood Transfusions; CM = Comorbidities; CHF = Chronic Heart Failure; COPD = Chronic Obstructive Pulmonary Disease; CPR = Cardiopulmonary Resuscitation; CVD = Cardiovascular Disease; ESRD = End-Stage Renal Disease; H = Hypothetical; ICD = Implantable Cardioverter Defibrillator; IV = Intravenous Fluids and Medication; LST = Life Sustaining Treatment; MM = Multimorbidity; MV = Mechanical Ventilation; *n* = number; NG = Nasogastric Tube Feeding; NIV = Non-Invasive Ventilation; PEG = Percutaneous Endoscopic Gastrostomy; R = Real; SD = Standard Deviation; T_1_ = Timepoint 1 (after intervention). * T_1_ values are presented, as baseline values were not reported; treatment preference stability was significantly higher in the intervention group. ^¥^ Summarized from the following answer categories: Refer to physician, Refer to family, Do not know, Blank. ^ž^ T_0_ values (baseline) are presented. ^§^ Yes (acceptors) summarizes all participants who would accept CPR/MV/IV/BT/NG in at least one of several (real and hypothetical) scenarios proposed, No (refusers) summarizes all participants that would not accept CPR in each of proposed scenarios. ^#^ Only “Older aged”, defined as 65 years or older, are included. ^£^ Having a “Do Not Resuscitate” order is interpreted as not wanting CPR (Answer category “No”).

**Table 4:** Other EoL care preferences.

Author, Year (Reference)	EoL Care Measure	Scenario	Type of Condition	Participants, *n*	Scenario Categories	Yes, *n* (%)	No, *n* (%)
Etkind, 2020 [30]	Extending life over relieving discomfort	R	MM (frail)	82			
Modes, 2019 [40]	Extending life over relieving discomfort	R & H	MM (median survival approx. 2 years, 18% cancer)	535	Relief > extension	321 (60)	-
					Extension > relief	91 (17)	
					Unsure	123 (23)	
Panocchia, 2017 [44]	Continuation of Dialysis (in at least one of 3 scenarios)	H	ESRD + CM	34	Dementia, terminal illness or coma	20 (59)	14 (41)
Tamura, 2010 [47]	Withdraw from dialysis	R & H	ESRD + CM	61	Current state of health	1 (2)	nr
					Moderate stroke	9 (15)	nr
					Dementia	20 (33)	nr
					Terminal cancer	20 (32)	nr
					Coma	36 (59)	nr

CM = Comorbidities; ESRD = End-Stage Renal Disease; H = Hypothetical; MM = Multimorbidity; *n* = number; nr = not reported; R = Real; SD = Standard Deviation.

**Table 5:** Preference for SDM.

Author, Year (Reference)	EoL Care Measure	Scenario	Type of Condition	Participants, *n*	Answer Categories	Yes, *n* (%)
**Who should be involved?**						
Agard, 2000 [26]	SDM related to CPR	R	CHF (NYHA II-IV) + CM	40	Patient	5 (12)
					Physician	15 (37)
					Both	18 (47)
Chan, 2007 [28]	SDM related to LST (non-specified)	H	MM	287	Patient	66 (23)
					Family	27 (9)
					Physician	107 (37)
					Depends	88 (31)
Panocchia, 2017 * [44]	SDM related to CPR, MV or PEG	H	ESRD + CM	34	Family	31 (91)
**Who should decide?**						
Chan, 2007 [28]	SDM related to LST (non-specified)	H	MM	287	Patient	48 (17)
					Family	15 (5)
					Physician	142 (50)
					Depends	82 (29)
Fuseya, 2019 [31]	SDM related to CPR/MV	R	COPD (severe) + CM	162	Patient	39 (24)/39 (24)
					Family	23 (14)/22 (14)
					Physician	71 (44)/68 (42)
					No answer/Do not know	27 (17)/31 (19)
Jerpseth, 2018 [37]	SDM related to MV or NIV	R	COPD (GOLD III-IV) + CM	12	Patient	10 (83)
					Physician	2 (17)
Naik, 2016 [41]	SDM related to EoL care	H	Advanced cancer + CM	146	Collaborative decision making (including family and physician)	32–146 (22–100) °
Nath, 2008 [42]	SDM related to EoL care	R	MM (Frail)	18	Patient	12–18 (60–100) °
Panocchia, 2017 * [44]	SDM related to CPR/MV/PEG	H	ESRD + CM	34	Family or friend only in case of incapability	25 (73)

CM = Comorbidities; CHF = Chronic Heart Failure; COPD = Chronic Obstructive Pulmonary Disease; CPR = Cardiopulmonary Resuscitation; EoL = End of Life; ESRD = End-Stage Renal Disease; H = Hypothetical; LST = Life Sustaining Treatment; MM = Multimorbidity; MV = Mechanical Ventilation; *n* = number; PEG = Percutaneous Endoscopic Gastrostomy; R = Real; SD = Standard Deviation; SDM = Shared Decision Making. * Multiple answers were possible. ° Verbal counts were transformed into numbers according to Chang et al. approach [25].

## Data Availability

Data is contained within the article or supplementary material.

## Supplementary Materials

The following are available online at https://www.mdpi.com/2077-0383/10/1/91/s1, Table S1: Preferred Reporting System Items for Systematic Review and Meta-Analysis (PRISMA) checklist, Table S2: Search for End-of-Life Care Preferences, Table S3: Excluded studies and reasons for exclusions, Table S4: Quality appraisal of included studies using the MMAT. Figure S1: PRISMA 2009 Flow Diagram.
