# Supplementary material for: End-of-Life Care Preferences of Older Patients with Multimorbidity: A Mixed Methods Systematic Review
Source: J Clin Med. 2020 Dec 29;10(1):91. doi: 10.3390/jcm10010091 (PMC7795676; doi:10.3390/jcm10010091)
Supplement: Supplementary file 1 [file jcm-10-00091-s001.zip › jcm-1043522-supplementary/Supplemental Table 4.docx]

**Supplemental Table 4. Risk of bias assessment of included studies using the Mixed Methods Appraisal Tool (MMAT)**

| **Author, Year (Reference)** | 1. **Qualitative studies** | | | | | **Framing effect** |
| --- | --- | --- | --- | --- | --- | --- |
|  | 1.1. Is the qualitative approach appropriate to answer the research question? | 1.2. Are the qualitative data collection methods suitable for addressing the research question? | 1.3. Are the findings adequately derived from the data? | 1.4. Is the interpretation of results sufficiently substantiated by data? | 1.5. Are qualitative data sources, collection, analysis and interpretation coherent? |  |
| Jerpseth, 2018 [43] | Yes | Yes | Yes | Yes | Yes | No |
| Naik, 2016 [41] | Yes | Yes | Yes | Yes | Yes | No |
| Strachan, 2011 [42] | Yes | Can’t tell | Yes | Yes | Yes | No |
| **Author, Year (Reference)** | 1. **Quantitative non-randomized studies** | | | | |  |
|  | 3.1. Are the participants representative of the target population? | 3.2. Are measurements appropriate to both the outcome and intervention (or exposure)? | 3.3. Are the outcome data complete? | 3.4. Are the confounders accounted for in the design and analysis? | 3.5 During the study period, was the intervention administered (or did exposure occur) as intended? |  |
| Chan, 2010 [47] | Yes | Yes | Yes | Yes | Yes | Yes, negative |
| **Author, Year (Reference)** | 1. **Quantitative descriptive studies** | | | | | **Framing effect** |
|  | 4.1. Is the sampling strategy relevant to address the research question? | 4.2. Is the sample representative of the target population? | 4.3. Are the measurements appropriate? | 4.4. Is the risk of non-response bias low? | 4.5. Is the statistical analysis appropriate to answer the research question? |  |
| Carlucci, 2016 [31] | Yes | Yes | Yes | No | Yes | No |
| Chan, 2007 [39] | Yes | Yes | Yes | Yes | Yes | No |
| Fuseya, 2019 [38] | Yes | Can’t tell | Can’t tell | No | Yes | Can’t tell |
| Houben, 2016 [36] | Yes | Yes | Yes | No | Yes | Yes, negative |
| Janssen, 2011 [35] | Yes | No | Yes | No | Yes | Yes, negative |
| Janssen, 2012 [34] | Yes | No | Yes | No | Yes | No |
| Janssen, 2013a [37] | Yes | No | Yes | No | Yes | No |
| Janssen, 2013b [33] | Yes | No | Yes | No | Yes | Yes, negative |
| Lee, 1992 [27] | Yes | No | Yes | No | Yes | No |
| Menon, 2000 [28] | Yes | No | Yes | No | Yes | No |
| Modes, 2019 [30] | Yes | Can’t tell | Yes | No | Yes | No |
| Ni, 2020 [40] | Yes | Yes | Yes | Yes | Yes | No |
| Panocchia, 2017 [32] | Yes | Can’t tell | Yes | No | Yes | No |
| Parr, 2010 [29] | Yes | Yes | Yes | Yes | Yes | No |
| Tamura, 2010 [26] | Yes | Yes | Yes | Yes | Yes | No |
| **Author, Year (Reference)** | 1. **Mixed methods** | | | | | **Framing effect** |
|  | 5.1. Is the rationale for using a mixed method design to address the research question convincing? | 5.2. Are the different components of the study effectively integrated? | 5.3. Are the outputs from the integration of qualitative and quantitative components adequately interpreted? | 5.4. Are divergences and inconsistencies between quantitative and qualitative results adequately addressed? | 5.5. Do the different components of the study adhere to the standard quality criteria for each of the methods involved? |  |
| Agard, 2000 [45] | Yes | Yes | Yes | Yes | Yes | No / Yes, positive |
| Etkind, 2020 | Yes | Yes | Yes | Yes | Yes | No |
| Nath, 2008 [44] | Yes | Yes | Yes | Yes | Yes | No |

Can’t tell = Author did not provide enough information for an assessment.
