# Supplementary material for: End-of-Life Care Preferences of Older Patients with Multimorbidity: A Mixed Methods Systematic Review
Source: J Clin Med. 2020 Dec 29;10(1):91. doi: 10.3390/jcm10010091 (PMC7795676; doi:10.3390/jcm10010091)
Supplement: Supplementary file 1 [file jcm-10-00091-s001.zip › jcm-1043522-supplementary/Supplementary Table 2. Search.docx]

**Table S2. Search for End-of-Life Care Preferences**

12.11.2020 – Medline via Ovid (medall)

|  | ((advanced OR incurabl* OR progressive OR life-limiting OR fatal OR serious* OR end-stage OR terminal*) adj3 (disease OR condition OR illness OR ill OR morbid*)).ti,ab,kf. | End-of-Life |
| --- | --- | --- |
|  | (End of life OR (last days adj3 life) OR (last year* adj3 life) OR (last week* adj3 life) OR (last month* adj3 life) OR (last days adj3 live) OR (last week* adj3 live) OR (last month* adj3 live) OR (last year* adj3 live) OR imminent death OR (close adj3 death) OR before death OR palliative).ti,ab,kf. | End-of-life |
|  | (Terminal Care OR Terminally Ill OR Hospice Care OR Life Support Care OR Advanced Cardiac Life Support OR Palliative Care).sh. |  |
|  | or/1-3 |  |
|  | (Comorbidity OR Multimorbidity OR Multiple Chronic Conditions).sh. | Multimorbidity |
|  | ((comorbid* OR multiple OR several OR multi OR concurrent OR complex OR more than one) adj4 (disease* OR condition* OR illness* OR morbid*)).ti,ab,kf. |  |
|  | (Comorbidit* OR multimorbidit* OR multidisease* OR polymorbid* OR frail*).ti,ab,kf. |  |
|  | or/5-7 |  |
|  | 4 AND 8 |  |
|  | (scale OR scaling OR ranking OR rating OR conjoint-analysis OR conjoint-analyses OR contingent valuation OR analytic hierarch* process* OR time trade off OR evidential reasoning OR multi-attribute utility OR maut OR multiattribute decision model OR madm OR electre iv OR electre is OR visual analog* scale OR score* OR scoring OR standard gamble OR EVIDEM OR paprika method OR simple additive weighting method OR weighted product method OR wpm OR technique for order preference by similarity to ideal solution OR topsis OR analytic network process OR anp OR todim OR macbeth OR smart OR focus group* OR interview* OR questionnair* OR choice).ti,ab,kf. | Methods to elicit Preferences |
|  | (prefer* OR wish* OR need OR needs OR value* OR belief* OR want* OR desire* OR priorit* OR attitude* OR perception* OR evaluation* OR choice* OR experience* OR decision* OR decide* O$R perspective*).ti,ab,kf. | Preferences |
|  | (patient* OR women* OR men* OR elder* OR old* OR frail*).ti,ab,kf. |  |
|  | 10 AND 11 AND 12 |  |
|  | (Patient Satisfaction OR Patient Preference OR Health Priorities OR Needs Assessment OR Advance Care Planning OR Advance Directives).sh. |  |
|  | 9 AND 13 |  |
|  | 9 AND 14 |  |
|  | or/15-16 | **2,492 articles retrieved** |
