# Supplementary material for: End-of-Life Care Preferences of Older Patients with Multimorbidity: A Mixed Methods Systematic Review
Source: J Clin Med. 2020 Dec 29;10(1):91. doi: 10.3390/jcm10010091 (PMC7795676; doi:10.3390/jcm10010091)
Supplement: Supplementary file 1 [file jcm-10-00091-s001.zip › jcm-1043522-supplementary/Supplementary Table 3.docx]

**Table S3. Excluded studies and reasons for exclusion**

| **Source** | **Reason for exclusion** |
| --- | --- |
| 1. (No authors listed). A controlled trial to improve care for seriously ill hospitalized patients. The study to understand prognoses and preferences for outcomes and risks of treatments (SUPPORT). The SUPPORT Principal Investigators. JAMA 1995; 274(20):1591–8. Available from: URL: https://www.cochranelibrary.com/central/doi/10.1002/central/CN-00120515/full. | Outcome: preferences not addressed |
| 2. Ahalt C, Walter LC, Yourman L, Eng C, Perez-Stable EJ, Smith AK. "Knowing is Better": Preferences of Diverse Older Adults for Discussing Prognosis. Journal of General Internal Medicine 2012; 27(5):568–75. Available from: URL: <Go to ISI>://WOS:000302869300017. | Outcome: preferences not addressed |
| 3. Anderson JE, Sikorski I, Finucane TE. Advance care planning by or on behalf of peritoneal dialysis patients in long-term care. American Journal of Kidney Diseases 2006; 48(1):122–7. Available from: URL: http://www.redi-bw.de/db/ebsco.php/search.ebscohost.com/login.aspx%3fdirect%3dtrue%26db%3dcin20%26AN%3d106185813%26site%3dehost-live. | Population: not multimorbid |
| 4. Auriemma CL, Nguyen CA, Bronheim R, Kent S, Nadiger S, Pardo D et al. Stability of End-of-Life Preferences A Systematic Review of the Evidence. Jama Internal Medicine 2014; 174(7):1085–92. Available from: URL: <Go to ISI>://WOS:000339491700019. | Document type: scoping or systematic reviews |
| 5. Barnato AE, Anthony DL, Skinner J, Gallagher PM, Fisher ES. Racial and Ethnic Differences in Preferences for End-of-Life Treatment. Journal of General Internal Medicine 2009; 24(6):695–701. Available from: URL: <Go to ISI>://WOS:000266241300001. | Population: not multimorbid |
| 6. Becerra M, Hurst SA, Perron NJ, Cochet S, Elger BS. ‘Do not attempt resuscitation’ and ‘cardiopulmonary resuscitation’ in an inpatient setting: Factors influencing physicians’ decisions in Switzerland. Gerontology 2011; 57(5):414–21. Available from: URL: http://www.redi-bw.de/db/ebsco.php/search.ebscohost.com/login.aspx%3fdirect%3dtrue%26db%3dpsyh%26AN%3d2011-19941-002%26site%3dehost-live. | Outcome: preferences from caregivers, family members and health care professionals |
| 7. Bekelman DB, Nowels CT, Retrum JH, Allen LA, Shakar S, Hutt E et al. Giving voice to patients' and family caregivers' needs in chronic heart failure: implications for palliative care programs. Journal of Palliative Medicine 2011; 14(12):1317–24. Available from: URL: http://ovidsp.ovid.com/ovidweb.cgi?T=JS&CSC=Y&NEWS=N&PAGE=fulltext&D=medc1&AN=22107107. | Outcome: preferences not addressed |
| 8. Bereza BG, Nielsen AT, Valgardsson S, Hemels MEH, Einarson TR. Patient preferences in severe COPD and asthma: a comprehensive literature review. International Journal of Chronic Obstructive Pulmonary Disease 2015; 10:739–44. Available from: URL: <Go to ISI>://WOS:000352381100003. | Document type: scoping or systematic reviews |
| 9. Bernacki RE, Block SD, Amer Coll Phys High Value, Care. Communication About Serious Illness Care Goals A Review and Synthesis of Best Practices. Jama Internal Medicine 2014; 174(12):1994–2003. Available from: URL: <Go to ISI>://WOS:000345911000027. | Document type: scoping or systematic reviews |
| 10. Bertolini G, Boffelli S, Malacarne P, Peta M, Marchesi M, Barbisan C et al. End-of-life decision-making and quality of ICU performance: an observational study in 84 Italian units. Intensive Care Medicine 2010; 36(9):1495–504. Available from: URL: http://www.redi-bw.de/db/ebsco.php/search.ebscohost.com/login.aspx%3fdirect%3dtrue%26db%3dcin20%26AN%3d104915575%26site%3dehost-live. | Outcome: preferences from caregivers, family members and health care professionals |
| 11. Bito S, Matsumura S, Singer MK, Meredith LS, Fukuhara S, Wenger NS. Acculturation and end-of-life decision making: comparison of Japanese and Japanese-American focus groups. Bioethics 2007; 21(5):251–62. Available from: URL: http://ovidsp.ovid.com/ovidweb.cgi?T=JS&CSC=Y&NEWS=N&PAGE=fulltext&D=med5&AN=17845470. | Population: not multimorbid |
| 12. Blackhall LJ, Frank G, Murphy ST, Michel V, Palmer JM, Azen SP. Ethnicity and attitudes towards life sustaining technology. Social Science & Medicine 1999; 48(12):1779–89. Available from: URL: <Go to ISI>://WOS:000080764500008. | Population: not multimorbid |
| 13. Bone AE, Morgan M, Maddocks M, Sleeman KE, Wright J, Taherzadeh S et al. Developing a model of short-term integrated palliative and supportive care for frail older people in community settings: perspectives of older people, carers and other key stakeholders. Age & Ageing 2016; 45(6):863–73. Available from: URL: http://ovidsp.ovid.com/ovidweb.cgi?T=JS&CSC=Y&NEWS=N&PAGE=fulltext&D=med12&AN=27586857. | Outcome: preferences from caregivers, family members and health care professionals |
| 14. Borum ML, Lynn J, Zhong ZS. The effects of patient race on outcomes in seriously ill patients in SUPPORT: An overview of economic impact, medical intervention, and end-of-life decisions. Journal of the American Geriatrics Society 2000; 48(5):S194-S198. Available from: URL: <Go to ISI>://WOS:000087033000026. | Document type: scoping or systematic reviews |
| 15. Bristowe K, Horsley HL, Shepherd K, Brown H, Carey I, Matthews B et al. Thinking ahead—The need for early advance care planning for people on haemodialysis: A qualitative interview study. Palliative Medicine 2015; 29(5):443–50. Available from: URL: http://www.redi-bw.de/db/ebsco.php/search.ebscohost.com/login.aspx%3fdirect%3dtrue%26db%3dpsyh%26AN%3d2015-18006-006%26site%3dehost-live. | Outcome: preferences not addressed |
| 16. Campos-Calderón C, Montoya-Juárez R, Hueso-Montoro C, Hernández-López E, Ojeda-Virto F, García-Caro MP. Interventions and decision-making at the end of life: the effect of establishing the terminal illness situation. BMC Palliative Care 2016; 15:1–11. Available from: URL: http://www.redi-bw.de/db/ebsco.php/search.ebscohost.com/login.aspx%3fdirect%3dtrue%26db%3dcin20%26AN%3d119345333%26site%3dehost-live. | Outcome: preferences from caregivers, family members and health care professionals |
| 17. Carmel S, Mutran EJ. Stability of elderly persons' expressed preferences regarding the use of life-sustaining treatments. Social Science & Medicine 1999; 49(3):303–11. Available from: URL: <Go to ISI>://WOS:000081203200002. | Population: not multimorbid |
| 18. Carrese JA, Mullaney JL, Faden RR, Finucane TE. Planning for death but not serious future illness: qualitative study of housebound elderly patients. British Medical Journal 2002; 325(7356):125–7. Available from: URL: <Go to ISI>://WOS:000177069900016. | Population: not multimorbid |
| 19. Cartwright J, Kayser-Jones J. End-of-life care in assisted living facilities: perceptions of residents, families, and staffs. Journal of Hospice & Palliative Nursing 2003; 5(3):143–51. Available from: URL: http://www.redi-bw.de/db/ebsco.php/search.ebscohost.com/login.aspx%3fdirect%3dtrue%26db%3dcin20%26AN%3d106715726%26site%3dehost-live. | Outcome: preferences not addressed |
| 20. Casarett D, van Ness PH, O'Leary JR, Fried TR. Are patient preferences for life-sustaining treatment really a barrier to hospice enrolment for older adults with serious illness? Journal of the American Geriatrics Society 2006; 54(3):472–8. Available from: URL: http://ovidsp.ovid.com/ovidweb.cgi?T=JS&CSC=Y&NEWS=N&PAGE=fulltext&D=med5&AN=16551315. | Population: not multimorbid |
| 21. Castle NG, Mor V. Advance care planning in nursing homes: Pre- and post-Patient Self-Determination Act. Health Services Research 1998; 33(1):101–24. Available from: URL: <Go to ISI>://WOS:000072969900008. | Outcome: preferences not addressed |
| 22. Chan HY, Pang SM. Readiness of Chinese frail old age home residents towards end-of-life care decision making. Journal of Clinical Nursing 2011; 20(9-10):1454–61. Available from: URL: http://ovidsp.ovid.com/ovidweb.cgi?T=JS&CSC=Y&NEWS=N&PAGE=fulltext&D=med7&AN=21492285. | Population: not multimorbid |
| 23. Chandna SM, Carpenter L, Da Silva-Gane M, Warwicker P, Greenwood RN, Farrington K. Rate of Decline of Kidney Function, Modality Choice, and Survival in Elderly Patients with Advanced Kidney Disease. Nephron 2016; 134(2):64–72. Available from: URL: http://ovidsp.ovid.com/ovidweb.cgi?T=JS&CSC=Y&NEWS=N&PAGE=fulltext&D=med12&AN=27423919. | Outcome: preferences not addressed |
| 24. Chen HB, Haley WE, Robinson BE, Schonwetter RS. Decisions for hospice care in patients with advanced cancer. Journal of the American Geriatrics Society 2003; 51(6):789–97. Available from: URL: <Go to ISI>://WOS:000183049200008. | Outcome: preferences not addressed |
| 25. Chikhladze N, Tebidze N, Chabukiani T, Chabukiani N, Chkhartishvili N, Jincharadze M et al. The Attitudes, Needs, and Requirements at End of Life in the Republic of Georgia (Comparative Analysis of Groups of Patients With Cancer and Elders). Journal of Palliative Care 2018; 33(4):252–9. Available from: URL: http://www.redi-bw.de/db/ebsco.php/search.ebscohost.com/login.aspx%3fdirect%3dtrue%26db%3dcin20%26AN%3d131860095%26site%3dehost-live. | Outcome: preferences not addressed |
| 26. Choi JY, Kim SW, Yoon SJ, Kang MG, Kim KI, Kim CH. Impact of frailty on do-not-resuscitate orders and healthcare transitions among elderly Koreans with pneumonia. Clinical Interventions In Aging 2018; 13:2237–45. Available from: URL: http://ovidsp.ovid.com/ovidweb.cgi?T=JS&CSC=Y&NEWS=N&PAGE=fulltext&D=medl&AN=30464432. | Outcome: preferences not addressed |
| 27. Chu L, Luk JK, Hui E, Chiu PK, Chan CS, Kwan F et al. Advance directive and end-of-life care preferences among chinese nursing home residents in hong kong. Journal of the American Medical Directors Association 2011; 12(2):143–52. Available from: URL: http://www.redi-bw.de/db/ebsco.php/search.ebscohost.com/login.aspx%3fdirect%3dtrue%26db%3dcin20%26AN%3d104991655%26site%3dehost-live. | Population: not multimorbid |
| 28. Cohen J, Bilsen J, Hooft P, Deboosere P, van der Wal G, Deliens L. Dying at home or in an institution using death certificates to explore the factors associated with place of death. Health Policy 2006; 78(2/3):319–29. Available from: URL: http://www.redi-bw.de/db/ebsco.php/search.ebscohost.com/login.aspx%3fdirect%3dtrue%26db%3dcin20%26AN%3d106253312%26site%3dehost-live. | Outcome: preferences not addressed |
| 29. Covinsky KE, Fuller JD, Yaffe K, Johnston CB, Hamel MB, Lynn J et al. Communication and decision-making in seriously ill patients: Findings of the SUPPORT project. Journal of the American Geriatrics Society 2000; 48(5):S187-S193. Available from: URL: <Go to ISI>://WOS:000087033000025. | Document type: scoping or systematic reviews |
| 30. Crump T, Llewellyn-Thomas HA. Assessing medicare beneficiaries' strength-of-preference scores for health care options: how engaging does the elicitation technique need to be? Health Expectations 2011; 14:33–45. Available from: URL: <Go to ISI>://WOS:000287412200004. | Population: not multimorbid |
| 31. Crump RT, Llewellyn-Thomas H. Characterizing the Public's Preferential Attitudes Toward End-of-Life Care Options: A Role for the Threshold Technique? Health Services Research 2013; 48(6):2101–24. Available from: URL: <Go to ISI>://WOS:000327392300017. | Population: not multimorbid |
| 32. Damghi N, Belayachi J, Aggoug B, Dendane T, Abidi K, Madani N et al. Withholding and withdrawing life-sustaining therapy in a Moroccan Emergency Department: an observational study. BMC Emergency Medicine 2011; 11:12. Available from: URL: http://ovidsp.ovid.com/ovidweb.cgi?T=JS&CSC=Y&NEWS=N&PAGE=fulltext&D=med7&AN=21838861. | Outcome: preferences from caregivers, family members and health care professionals |
| 33. Danis M, Garrett J, Harris R, Patrick DL. STABILITY OF CHOICES ABOUT LIFE-SUSTAINING TREATMENTS. Annals of Internal Medicine 1994; 120(7):567–73. Available from: URL: <Go to ISI>://WOS:A1994NC76400006. | Outcome: preferences from caregivers, family members and health care professionals |
| 34. Danis M, Patrick DL, Southerland LI, Green ML. PATIENTS AND FAMILIES PREFERENCES FOR MEDICAL INTENSIVE-CARE. Jama-Journal of the American Medical Association 1988; 260(6):797–802. Available from: URL: <Go to ISI>://WOS:A1988P523200025. | Population: not multimorbid |
| 35. Davison SN. End-of-Life Care Preferences and Needs: Perceptions of Patients with Chronic Kidney Disease. Clinical Journal of the American Society of Nephrology 2010; 5(2):195–204. Available from: URL: <Go to ISI>://WOS:000274703400006. | Population: not multimorbid |
| 36. Deschepper R, Bernheim JL, Vander Stichele R, van den Block L, Michiels E, van der Kelen G et al. Truth-telling at the end of life: a pilot study on the perspective of patients and professional caregivers. Patient Education & Counseling 2008; 71(1):52–6. Available from: URL: http://www.redi-bw.de/db/ebsco.php/search.ebscohost.com/login.aspx%3fdirect%3dtrue%26db%3dcin20%26AN%3d105754355%26site%3dehost-live. | Outcome: preferences not addressed |
| 37. Ditto PH, Jacobson JA, Smucker WD, Danks JH, Fagerlin A. Context Changes Choices: A Prospective Study of the Effects of Hospitalization on Life-Sustaining Treatment Preferences. Medical Decision Making 2006; 26(4):313–22. Available from: URL: http://www.redi-bw.de/db/ebsco.php/search.ebscohost.com/login.aspx%3fdirect%3dtrue%26db%3dpsyh%26AN%3d2006-09437-004%26site%3dehost-live. | Population: not multimorbid |
| 38. Ditto PH, Smucker WD, Danks JH, Jacobson JA, Houts RM, Fagerlin A et al. Stability of older adults' preferences for life-sustaining medical treatment. Health Psychology 2003; 22(6):605–15. Available from: URL: <Go to ISI>://WOS:000186863700008. | Population: not multimorbid |
| 39. Downar J, Luk T, Sibbald RW, Santini T, Mikhael J, Berman H et al. Why do patients agree to a 'Do Not Resuscitate' or 'full code' order? Perspectives of medical inpatients. Journal of General Internal Medicine 2011; 26(6):582–7. Available from: URL: http://www.redi-bw.de/db/ebsco.php/search.ebscohost.com/login.aspx%3fdirect%3dtrue%26db%3dpsyh%26AN%3d2011-10240-007%26site%3dehost-live. | Population: not multimorbid |
| 40. Downey L, Au DH, Curtis JR, Engelberg RA. Life-Sustaining Treatment Preferences: Matches and Mismatches Between Patients' Preferences and Clinicians' Perceptions. Journal of Pain and Symptom Management 2013; 46(1):9–19. Available from: URL: <Go to ISI>://WOS:000321748500002. | Outcome: preferences from caregivers, family members and health care professionals |
| 41. Eleazer GP, Hornung CA, Egbert CB, Egbert JR, Eng C, Hedgepeth J et al. The relationship between ethnicity and advance directives in a frail older population. Journal of the American Geriatrics Society 1996; 44(8):938–43. Available from: URL: <Go to ISI>://WOS:A1996VA56300008. | Outcome: preferences not addressed |
| 42. Elkin EB, Kim SHM, Casper ES, Kissane DW, Schrag D. Desire for information and involvement in treatment decisions: Elderly cancer patients' preferences and their physicians' perceptions. Journal of Clinical Oncology 2007; 25(33):5275–80. Available from: URL: <Go to ISI>://WOS:000251074400022. | Population: not multimorbid |
| 43. Emanuel LL, Barry MJ, Emanuel EJ, Stoeckle JD. ADVANCE DIRECTIVES - CAN PATIENTS STATED TREATMENT CHOICES BE USED TO INFER UNSTATED CHOICES. Medical Care 1994; 32(2):95–105. Available from: URL: <Go to ISI>://WOS:A1994MV78500001. | Population: age < 60 yrs. |
| 44. Eng C. Future consideration for improving end-of-life care for older persons: Program of All-inclusive Care for the Elderly (PACE). Journal of Palliative Medicine 2002; 5(2):305–10. Available from: URL: <Go to ISI>://MEDLINE:12013013. | Document type: editorials, narrative reviews |
| 45. Enguidanos S, Ailshire J. Timing of Advance Directive Completion and Relationship to Care Preferences. Journal of Pain and Symptom Management 2017; 53(1):49–56. Available from: URL: <Go to ISI>://WOS:000396502600009. | Population: not multimorbid |
| 46. Esteve A, Jimenez C, Perez R, Gomez JA. Factors related to withholding life-sustaining treatment in hospitalized elders. Journal of Nutrition, Health & Aging 2009; 13(7):644–50. Available from: URL: http://ovidsp.ovid.com/ovidweb.cgi?T=JS&CSC=Y&NEWS=N&PAGE=fulltext&D=med6&AN=19621201. | Outcome: preferences from caregivers, family members and health care professionals |
| 47. Everhart MA, Pearlman RA. STABILITY OF PATIENT PREFERENCES REGARDING LIFE-SUSTAINING TREATMENTS. Chest 1990; 97(1):159–64. Available from: URL: <Go to ISI>://WOS:A1990CG29200033. | Population: not multimorbid |
| 48. Fairrow AM, McCallum TJ, Messinger-Rapport BJ. Preferences of older African-Americans for long-term tube feeding at the end of life. Aging & Mental Health 2004; 8(6):530–4. Available from: URL: <Go to ISI>://WOS:000225405300007. | Population: not multimorbid |
| 49. Fakhri S, Engelberg RA, Downey L, Nielsen EL, Paul S, Lahdya AZ et al. Factors Affecting Patients' Preferences for and Actual Discussions About End-of-Life Care. Journal of Pain and Symptom Management 2016; 52(3):386–94. Available from: URL: <Go to ISI>://WOS:000386830500012. | Outcome: preferences not addressed |
| 50. Finkelstein EA, Bilger M, Flynn TN, Malhotra C. Preferences for end-of-life care among community-dwelling older adults and patients with advanced cancer: A discrete choice experiment. Health Policy 2015; 119(11):1482–9. Available from: URL: http://www.redi-bw.de/db/ebsco.php/search.ebscohost.com/login.aspx%3fdirect%3dtrue%26db%3dcin20%26AN%3d111184047%26site%3dehost-live. | Population: not multimorbid |
| 51. Finkelstein EA, Ozdemir S, Malhotra C, Jafar TH, Choong Hui Lin L, Gan Shien Wen S. Understanding factors that influence the demand for dialysis among elderly in a multi-ethnic Asian society. Health Policy 2018; 122(8):915–21. Available from: URL: http://ovidsp.ovid.com/ovidweb.cgi?T=JS&CSC=Y&NEWS=N&PAGE=fulltext&D=medl&AN=30007521. | Outcome: preferences not addressed |
| 52. Fusi-Schmidhauser T, Froggatt K, Preston N. Living with Advanced Chronic Obstructive Pulmonary Disease: A Qualitative Interview Study with Patients and Informal Carers. Copd. 2020;17(4):410-418. | Population: not multimorbid |
| 53. Fluur C, Bolse K, Stromberg A, Thylen I. Patients' experiences of the implantable cardioverter defibrillator (ICD) with a focus on battery replacement and end-of-life issues. Heart & Lung 2013; 42(3):202–7. Available from: URL: <Go to ISI>://WOS:000318891000008. | Population: not multimorbid |
| 54. Flynn TN, Bilger M, Malhotra C, Finkelstein EA. Are Efficient Designs Used in Discrete Choice Experiments Too Difficult for Some Respondents? A Case Study Eliciting Preferences for End-of-Life Care. Pharmacoeconomics 2016; 34(3):273–84. Available from: URL: <Go to ISI>://WOS:000371164200007. | Population: not multimorbid |
| 55. Fraser SM, Torres GL, Cai C, Choi HA, Sharrief A, Chang TR. Race is a Predictor of Withdrawal of Life Support in Patients with Intracerebral Hemorrhage. Journal of Stroke & Cerebrovascular Diseases 2018; 27(11):3108–14. Available from: URL: http://ovidsp.ovid.com/ovidweb.cgi?T=JS&CSC=Y&NEWS=N&PAGE=fulltext&D=med13&AN=30121154. | Population: not multimorbid |
| 56. Fried TR, Bradley EH. What Matters to Seriously Ill Older Persons Making End-of-Life Treatment Decisions?: A Qualitative Study. Journal of Palliative Medicine 2003; 6(2):237–44. Available from: URL: http://www.redi-bw.de/db/ebsco.php/search.ebscohost.com/login.aspx%3fdirect%3dtrue%26db%3dpsyh%26AN%3d2006-07168-008%26site%3dehost-live. | Population: not multimorbid |
| 57. Fried TR, Bradley EH, Towle VR. Assessment of patient preferences: Integrating treatments and outcomes. Journals of Gerontology Series B-Psychological Sciences and Social Sciences 2002; 57(6):S348-S354. Available from: URL: <Go to ISI>://WOS:000179112000016. | Population: not multimorbid |
| 58. Fried TR, Bradley EH, Towle VR, Allore H. Understanding the treatment preferences of seriously ill patients. New England Journal of Medicine 2002; 346(14):1061–6. Available from: URL: <Go to ISI>://WOS:000174729400007. | Population: not multimorbid |
| 59. Fried TR, Byers AL, Gallo WT, van Ness PH, Towle VR, O'Leary JR et al. Prospective study of health status preferences and changes in preferences over time in older adults. Archives of Internal Medicine 2006; 166(8):890–5. Available from: URL: <Go to ISI>://WOS:000237026000009. | Population: not multimorbid |
| 60. Fried TR, McGraw S, Agostini JV, Tinetti ME. Views of older persons with multiple morbidities on competing outcomes and clinical decision-making. Journal of the American Geriatrics Society 2008; 56(10):1839–44. Available from: URL: <Go to ISI>://WOS:000259900100009. | Outcome: preferences not addressed |
| 61. Fried TR, O'Leary J, van Ness P, Fraenkel L. Inconsistency over time in the preferences of older persons with advanced illness for life-sustaining treatment. Journal of the American Geriatrics Society 2007; 55(7):1007–14. Available from: URL: <Go to ISI>://WOS:000247607100005. | Population: not multimorbid |
| 62. Fried TR, Tinetti ME, Iannone L, O'Leary JR, Towle V, van Ness PH. Health Outcome Prioritization as a Tool for Decision Making Among Older Persons With Multiple Chronic Conditions. Archives of Internal Medicine 2011; 171(20):1854–6. Available from: URL: <Go to ISI>://WOS:000297056300016. | Outcome: preferences not addressed |
| 63. Fried TR, Tinetti M, Agostini J, Lannone L, Towle V. Health outcome prioritization to elicit preferences of older persons with multiple health conditions. Patient Education and Counseling 2011; 83(2):278–82. Available from: URL: <Go to ISI>://WOS:000290929200026. | Outcome: preferences not addressed |
| 64. Fried TR, van Ness PH, Byers AL, Towle VR, O'Leary JR, Dubin JA. Changes in preferences for life-sustaining treatment among older persons with advanced illness. Journal of General Internal Medicine 2007; 22(4):495–501. Available from: URL: <Go to ISI>://WOS:000245107800012. | Population: not multimorbid |
| 65. Friend JM, Alden DL. Improving Patient Preparedness and Confidence in Discussing Advance Directives for End-of-Life Care with Health Care Providers in the United States and Japan. Med Decis Making. 2020:272989x20969683. | Population: age < 60 yrs. |
| 66. Gehlbach TG, Shinkunas LA, Forman-Hoffman VL, Thomas KW, Schmidt GA, Kaldjian LC. Code Status Orders and Goals of Care in the Medical ICU. Chest 2011; 139(4):802–9. Available from: URL: <Go to ISI>://WOS:000289538500013. | Population: not multimorbid |
| 67. Gheorghe C, Vazquez R, Casanegra AI, Argento V, Dadu R, Feng Y et al. Elders' Environs and Their End-Of-Life Preferences. Journal of the American Medical Directors Association 2011; 12(1):22–8. Available from: URL: <Go to ISI>://WOS:000286550900006. | Population: not multimorbid |
| 68. Girones R, Torregrosa D, Gomez-Codina J, Maestu I, Tenias JM, Rosell R. Lung cancer chemotherapy decisions in older patients: the role of patient preference and interactions with physicians. Clinical & Translational Oncology: Official Publication of the Federation of Spanish Oncology Societes & of the National Cancer Institute of Mexico 2012; 14(3):183–9. Available from: URL: http://ovidsp.ovid.com/ovidweb.cgi?T=JS&CSC=Y&NEWS=N&PAGE=fulltext&D=med8&AN=22374421. | Outcome: preferences not addressed |
| 69. Golin CE, Wenger NS, Liu HH, Dawson NV, Teno JM, Desbiens NA et al. A prospective study of patient-physician communication about resuscitation. Journal of the American Geriatrics Society 2000; 48(5):S52-S60. Available from: URL: <Go to ISI>://WOS:000087033000008. | Population: age < 60 yrs. |
| 70. Graabæk T, Lundby C, Ryg J, Søndergaard J, Pottegård A, Nielsen DS. “I simply don't know, because I don't know which drugs I get”: Perspectives on deprescribing among older adults with limited life expectancy and their relatives. Basic & Clinical Pharmacology & Toxicology.n/a(n/a). | Population: not multimorbid |
| 71. Graw JA, Spies CD, Wernecke KD, Braun JP. Managing end-of-life decision making in intensive care medicine--a perspective from Charite Hospital, Germany. PLoS ONE [Electronic Resource] 2012; 7(10):e46446. Available from: URL: http://ovidsp.ovid.com/ovidweb.cgi?T=JS&CSC=Y&NEWS=N&PAGE=fulltext&D=med8&AN=23049701. | Population: not multimorbid |
| 72. Gready RM, Ditto PH, Danks JH, Coppola KM, Lockhart LK, Smucker WD. Actual and perceived stability of preferences for life-sustaining treatment. Journal of Clinical Ethics 2000; 11(4):334–46. Available from: URL: <Go to ISI>://WOS:000167307500008. | Population: not multimorbid |
| 73. Guentner K, Hoffman LA, Happ MB, Kim Y, Dabbs A, Mendelsohn AB et al. Preferences for mechanical ventilation among survivors of prolonged mechanical ventilation and tracheostomy. American Journal of Critical Care 2006; 15(1):65–77. Available from: URL: <Go to ISI>://WOS:000202978500011. | Population: not multimorbid |
| 74. Guidet B, Flaatten H, Boumendil A, Morandi A, Andersen FH, Artigas A et al. Withholding or withdrawing of life-sustaining therapy in older adults (>= 80 years) admitted to the intensive care unit. Intensive Care Medicine 2018; 44(7):1027–38. Available from: URL: http://ovidsp.ovid.com/ovidweb.cgi?T=JS&CSC=Y&NEWS=N&PAGE=fulltext&D=medc1&AN=29774388. | Outcome: preferences from caregivers, family members and health care professionals |
| 75. Haidet P, Hamel MB, Davis RB, Wenger N, Reding D, Kussin PS et al. Outcomes, preferences for resuscitation, and physician-patient communication among patients with metastatic colorectal cancer. American Journal of Medicine 1998; 105(3):222–9. Available from: URL: <Go to ISI>://WOS:000075982200008. | Population: not multimorbid |
| 76. Happ MB, Capezuti E, Strumpf NE, Wagner L, Cunningham S, Evans L et al. Advance care planning and end-of-life care for hospitalized nursing home residents. Journal of the American Geriatrics Society 2002; 50(5):829–35. Available from: URL: http://ovidsp.ovid.com/ovidweb.cgi?T=JS&CSC=Y&NEWS=N&PAGE=fulltext&D=medc&AN=12028168. | Outcome: preferences from caregivers, family members and health care professionals |
| 77. Haydar ZR, Lowe AJ, Kahveci KL, Weatherford W, Finucane T. Differences in end-of-life preferences between congestive heart failure and dementia in a medical house calls program. Journal of the American Geriatrics Society 2004; 52(5):736–40. Available from: URL: http://www.redi-bw.de/db/ebsco.php/search.ebscohost.com/login.aspx%3fdirect%3dtrue%26db%3dcin20%26AN%3d106667215%26site%3dehost-live. | Outcome: preferences from caregivers, family members and health care professionals |
| 78. Heyland DK, Frank C, Groll D, Pichora D, Dodek P, Rocker G et al. Understanding cardiopulmonary resuscitation decision making - Perspectives of seriously ill hospitalized patients and family members. Chest 2006; 130(2):419–28. Available from: URL: <Go to ISI>://WOS:000239886800018. | Population: not multimorbid |
| 79. Heyland DK, Groll D, Rocker G, Dodek P, Gafni A, Tranmer J et al. End-of-life care in acute care hospitals in Canada: a quality finish? Journal of Palliative Care 2005; 21(3):142–50. Available from: URL: http://www.redi-bw.de/db/ebsco.php/search.ebscohost.com/login.aspx%3fdirect%3dtrue%26db%3dcin20%26AN%3d106550713%26site%3dehost-live. | Outcome: preferences not addressed |
| 80. Heyland DK, Heyland R, Dodek P, You JJ, Sinuff T, Hiebert T et al. Discordance between patients' stated values and treatment preferences for end-of-life care: results of a multicentre survey. Bmj Supportive & Palliative Care 2017; 7(3):292–9. Available from: URL: <Go to ISI>://WOS:000427549100015. | Population: not multimorbid |
| 81. Hong M, Kim K. Advance care planning among ethnic/racial minority older adults: Prevalence of and factors associated with informal talks, durable power of attorney for health care, and living will. Ethn Health. 2020:1-10. | Outcome: preferences not addressed |
| 82. Houben CHM, Spruit MA, Groenen MTJ, Wouters EFM, Janssen DJA. Efficacy of Advance Care Planning: A Systematic Review and Meta-Analysis. Journal of the American Medical Directors Association 2014; 15(7):477–89. Available from: URL: <Go to ISI>://WOS:000341166300005. | Document type: scoping or systematic reviews |
| 83. Houben CHM, Spruit MA, Schols J, Wouters EFM, Janssen DJA. Patient-Clinician Communication About End-of-Life Care in Patients With Advanced Chronic Organ Failure During One Year. Journal of Pain and Symptom Management 2015; 49(6):1109–15. Available from: URL: <Go to ISI>://WOS:000356604700021. | Outcome: preferences not addressed |
| 84. Houts RM, Smucker WD, Jacobson JA, Ditto PH, Danks JH. Predicting elderly outpatient's life-sustaining treatment preferences over time: The majority rules. Medical Decision Making 2002; 22(1):39–52. Available from: URL: http://www.redi-bw.de/db/ebsco.php/search.ebscohost.com/login.aspx%3fdirect%3dtrue%26db%3dpsyh%26AN%3d2002-00380-004%26site%3dehost-live. | Population: not multimorbid |
| 85. Howard M, Bansback N, Tan A, Klein D, Bernard C, Barwich D et al. Recognizing difficult trade-offs: values and treatment perferences for end-of-life care in a multi-site survey of adult patients in family practices. Bmc Medical Informatics and Decision Making 2017; 17. Available from: URL: <Go to ISI>://WOS:000417556700006. | Population: not multimorbid |
| 86. Howard M, Bansback N, Tan A, Klein D, Bernard C, Barwich D et al. Recognizing difficult trade-offs: values and treatment preferences for end-of-life care in a multi-site survey of adult patients in family practices. BMC Medical Informatics & Decision Making 2017; 17:1–9. Available from: URL: http://www.redi-bw.de/db/ebsco.php/search.ebscohost.com/login.aspx%3fdirect%3dtrue%26db%3dcin20%26AN%3d127005705%26site%3dehost-live. | Population: not multimorbid |
| 87. Hwang IC, Keam B, Yun YH, Ahn HY, Kim YA. Quality of life changes and intensive care preferences in terminal cancer patients. Palliative & Supportive Care 2015; 13(5):1309–16. Available from: URL: <Go to ISI>://WOS:000365663900017. | Population: not multimorbid |
| 88. Hwang IC, Keam B, Kim YA, Yun YH. Factors Related to the Differential Preference for Cardiopulmonary Resuscitation Between Patients With Terminal Cancer and That of Their Respective Family Caregivers. American Journal of Hospice & Palliative Medicine 2016; 33(1):20–6. Available from: URL: <Go to ISI>://WOS:000367725300004. | Outcome: preferences not addressed |
| 89. Ingravallo F, Mignani V, Mariani E, Ottoboni G, Melon MC, Chattat R. Discussing advance care planning: insights from older people living in nursing homes and from family members. International Psychogeriatrics 2018; 30(4):569–79. Available from: URL: http://ovidsp.ovid.com/ovidweb.cgi?T=JS&CSC=Y&NEWS=N&PAGE=fulltext&D=medl&AN=28988561. | Population: not multimorbid |
| 90. Isaac ML, Curtis JR, Pisani M. Addressing End-of-Life Issues in Older Patients with Lung Disease. Aging and Lung Disease: A Clinical Guide 2012:283–303. Available from: URL: <Go to ISI>://WOS:000303704000015. | Document type: editorials, narrative reviews |
| 91. Jabbarian LJ, Maciejewski RC, Maciejewski PK, Rietjens JAC, Korfage IJ, van der Heide A et al. The Stability of Treatment Preferences Among Patients With Advanced Cancer. Journal of Pain and Symptom Management 2019; 57(6):1071-+. Available from: URL: <Go to ISI>://WOS:000468425500011. | Population: not multimorbid |
| 92. Jager KJ, Korevaar JC, Dekker FW, Krediet RT, Boeschoten EW, Necosad Study Grp. The effect of contraindications and patient preference on dialysis modality selection in ESRD patients in The Netherlands. American Journal of Kidney Diseases 2004; 43(5):891–9. Available from: URL: <Go to ISI>://WOS:000221104800015. | Outcome: preferences not addressed |
| 93. Jayanti A, Neuvonen M, Wearden A, Morris J, Foden P, Brenchley P et al. Healthcare decision-making in end stage renal disease-patient preferences and clinical correlates. BMC Nephrology 2015; 16:17. Available from: URL: <Go to ISI>://WOS:000364804400001. | Outcome: preferences not addressed |
| 94. Johnston BM, Milligan S, Foster C, Kearney N. Self-care and end of life care-patients' and carers' experience a qualitative study utilising serial triangulated interviews. Supportive Care in Cancer 2012; 20(8):1619–27. Available from: URL: <Go to ISI>://WOS:000306129600004. | Outcome: preferences not addressed |
| 95. Jones J, Nowels C, Kutner JS, Matlock DD. Shared decision making and the use of a patient decision aid in advanced serious illness: provider and patient perspectives. Health Expectations 2015; 18(6):3236–47. Available from: URL: http://ovidsp.ovid.com/ovidweb.cgi?T=JS&CSC=Y&NEWS=N&PAGE=fulltext&D=med11&AN=25439268. | Outcome: preferences not addressed |
| 96. Kaldjian LC, Erekson ZD, Haberle TH, Curtis AE, Shinkunas LA, Cannon KT et al. Code status discussions and goals of care among hospitalised adults. Journal of Medical Ethics 2009; 35(6):338–42. Available from: URL: <Go to ISI>://WOS:000266453900001. | Outcome: preferences not addressed |
| 97. Kamar FB, Tam-Tham H, Thomas C. A Description of Advanced Chronic Kidney Disease Patients in a Major Urban Center Receiving Conservative Care. Canadian Journal of Kidney Health & Disease 2017; 4:2054358117718538. Available from: URL: http://ovidsp.ovid.com/ovidweb.cgi?T=JS&CSC=Y&NEWS=N&PAGE=fulltext&D=prem2&AN=28835848. | Outcome: preferences not addressed |
| 98. Kernerman P, Cook DJ, Griffith LE. Documenting life-support preferences in hospitalized patients. Journal of Critical Care 1997; 12(4):155–60. Available from: URL: http://www.redi-bw.de/db/ebsco.php/search.ebscohost.com/login.aspx%3fdirect%3dtrue%26db%3dcin20%26AN%3d107296795%26site%3dehost-live. | Outcome: preferences not addressed |
| 99. Kim YS, Escobar GJ, Halpern SD, Greene JD, Kipnis P, Liu V. The natural history of changes in preferences for life-sustaining treatments and implications for inpatient mortality in younger and older hospitalized adults. Journal of the American Geriatrics Society 2016; 64(5):981–9. Available from: URL: http://www.redi-bw.de/db/ebsco.php/search.ebscohost.com/login.aspx%3fdirect%3dtrue%26db%3dpsyh%26AN%3d2016-26347-011%26site%3dehost-live. | Population: not multimorbid |
| 100. Klemmt M, Henking T, Heizmann E, Best L, van Oorschot B, Neuderth S. Wishes and needs of nursing home residents and their relatives regarding end-of-life decision-making and care planning—A qualitative study. Journal of Clinical Nursing. 2020;29(13-14):2663-2674. | Population: age < 60 yrs. |
| 101. Ko E, Lee J. End-Of-Life Treatment Preference among Low-Income Older Adults: A Race/Ethnicity Comparison Study. Journal of Health Care for the Poor and Underserved 2014; 25(3):1021–33. Available from: URL: <Go to ISI>://WOS:000340306300007. | Population: not multimorbid |
| 102. Kobza R, Erne P. End-of-life decisions in ICD patients with malignant tumors. Pace-Pacing and Clinical Electrophysiology 2007; 30(7):845–9. Available from: URL: <Go to ISI>://WOS:000247856800004. | Population: not multimorbid |
| 103. Krumholz HM, Phillips RS, Hamel MB, Teno JM, Bellamy P, Broste SK et al. Resuscitation preferences among patients with severe congestive heart failure - Results from the SUPPORT project. Circulation 1998; 98(7):648–55. Available from: URL: <Go to ISI>://WOS:000075348600007. | Population: age < 60 yrs. |
| 104. Kwak J, Haley WE. Current research findings on end-of-life decision making among racially or ethnically diverse groups. Gerontologist 2005; 45(5):634–41. Available from: URL: <Go to ISI>://WOS:000232251900008. | Document type: scoping or systematic reviews |
| 105. Kwak J, Ellis JL. Factors related to advance care planning among older African American women: Age, medication, and acute care visits. Palliat Support Care. 2020;18(4):413-418. | Outcome: preferences not addressed |
| 106. Laakkonen ML, Pitkala KH, Strandberg TE, Berglind S, Tilvis RS. Living will, resuscitation preferences, and attitudes towards life in an aged population. Gerontology 2004; 50(4):247–54. Available from: URL: <Go to ISI>://WOS:000222476600009. | Population: not multimorbid |
| 107. Laakkonen ML, Pitkala KH, Strandberg TE. Terminally ill elderly patient's experiences, attitudes, and needs: A qualitative study. Omega-Journal of Death and Dying 2004; 49(2):117–29. Available from: URL: <Go to ISI>://WOS:000224110800004. | Outcome: preferences not addressed |
| 108. Lee M, Ganzini L. THE EFFECT OF RECOVERY FROM DEPRESSION ON PREFERENCES FOR LIFE-SUSTAINING THERAPY IN OLDER PATIENTS. Journals of Gerontology 1994; 49(1):M15-M21. Available from: URL: <Go to ISI>://WOS:A1994MQ84500014. | Population: not multimorbid |
| 109. Lee W-J, Peng L-N, Lin C-H, et al. First insights on value-based healthcare of elders using ICHOM older person standard set reporting. BMC Geriatrics. 2020;20(1):335. | Population: not multimorbid |
| 110. Levenson JW, McCarthy EP, Lynn J, Davis RB, Phillips RS. The last six months of life for patients with congestive heart failure. Journal of the American Geriatrics Society 2000; 48(5):S101-S109. Available from: URL: <Go to ISI>://WOS:000087033000014. | Outcome: preferences from caregivers, family members and health care professionals |
| 111. Levy C, Ersek M, Scott W, et al. Life-Sustaining Treatment Decisions Initiative: Early Implementation Results of a National Veterans Affairs Program to Honor Veterans' Care Preferences. J Gen Intern Med. 2020;35(6):1803-1812. | Population: not multimorbid |
| 112. Lin CP, Peng JK, Chen PJ, Huang HL, Hsu SH, Cheng SY. Preferences on the Timing of Initiating Advance Care Planning and Withdrawing Life-Sustaining Treatment between Terminally-Ill Cancer Patients and Their Main Family Caregivers: A Prospective Study. Int J Environ Res Public Health. 2020;17(21). | Population: not multimorbid |
| 113. Liu LN, Chen CH, Liu TW, Lin YC, Lee SC, Tang ST. Preferences for Aggressive End-of-life Care and Their Determinants Among Taiwanese Terminally Ill Cancer Patients. Cancer Nursing 2015; 38(3):E9-E18. Available from: URL: http://ovidsp.ovid.com/ovidweb.cgi?T=JS&CSC=Y&NEWS=N&PAGE=fulltext&D=med11&AN=24915466. | Population: not multimorbid |
| 114. Lloyd A, Kendall M, Starr JM, Murray SA. Physical, social, psychological and existential trajectories of loss and adaptation towards the end of life for older people living with frailty: a serial interview study. BMC Geriatrics 2016; 16(1):176. Available from: URL: http://ovidsp.ovid.com/ovidweb.cgi?T=JS&CSC=Y&NEWS=N&PAGE=fulltext&D=med12&AN=27765011. | Outcome: preferences not addressed |
| 115. Lo Y, Wang J, Liu L, Wang C. Prevalence and Related Factors of Do-Not-Resuscitate Directives Among Nursing Home Residents in Taiwan. Journal of the American Medical Directors Association 2010; 11(6):436–42. Available from: URL: http://www.redi-bw.de/db/ebsco.php/search.ebscohost.com/login.aspx%3fdirect%3dtrue%26db%3dcin20%26AN%3d104918886%26site%3dehost-live. | Outcome: preferences not addressed |
| 116. Lovell S, Walker RJ, Schollum JB, Marshall MR, McNoe BM, Derrett S. To dialyse or delay: a qualitative study of older New Zealanders' perceptions and experiences of decision-making, with stage 5 chronic kidney disease. BMJ Open 2017; 7(3):e014781. Available from: URL: http://ovidsp.ovid.com/ovidweb.cgi?T=JS&CSC=Y&NEWS=N&PAGE=fulltext&D=medc1&AN=28360253. | Outcome: preferences not addressed |
| 117. Lynn J, Ely EW, Zhong ZS, McNiff KL, Dawson NV, Connors A et al. Living and dying with chronic obstructive pulmonary disease. Journal of the American Geriatrics Society 2000; 48(5):S91-S100. Available from: URL: <Go to ISI>://WOS:000087033000013. | Outcome: preferences from caregivers, family members and health care professionals |
| 118. Maciejewski PK, Prigerson HG. Emotional numbness modifies the effect of end-of-life discussions on end-of-life care. Journal of Pain & Symptom Management 2013; 45(5):841–7. Available from: URL: http://ovidsp.ovid.com/ovidweb.cgi?T=JS&CSC=Y&NEWS=N&PAGE=fulltext&D=med9&AN=22926093. | Population: age < 60 yrs. |
| 119. Mack JW, Weeks JC, Wright AA, Block SD, Prigerson HG. End-of-Life Discussions, Goal Attainment, and Distress at the End of Life: Predictors and Outcomes of Receipt of Care Consistent With Preferences. Journal of Clinical Oncology 2010; 28(7):1203–8. Available from: URL: <Go to ISI>://WOS:000274892500021. | Population: age < 60 yrs. |
| 120. Maida V, Peck J, Ennis M, Brar N, Maida AR. Preferences for active and aggressive intervention among patients with advanced cancer. BMC Cancer 2010; 10:592. Available from: URL: http://ovidsp.ovid.com/ovidweb.cgi?T=JS&CSC=Y&NEWS=N&PAGE=fulltext&D=med7&AN=21029455. | Population: not multimorbid |
| 121. Maldonado LY, Goodson RB, Mulroy MC, Johnson EM, Reilly JM, Homeier DC. Wellness in Sickness and Health (The WISH Project): Advance Care Planning Preferences and Experiences Among Elderly Latino Patients. Clinical Gerontologist 2019; 42(3):259–66. Available from: URL: <Go to ISI>://WOS:000467730800005. | Population: not multimorbid |
| 122. Malia C, Bennett MI. What influences patients’ decisions on artificial hydration at the end of life? A Q-methodology study. Journal of Pain and Symptom Management 2011; 42(2):192–201. Available from: URL: http://www.redi-bw.de/db/ebsco.php/search.ebscohost.com/login.aspx%3fdirect%3dtrue%26db%3dpsyh%26AN%3d2011-17424-003%26site%3dehost-live. | Population: not multimorbid |
| 123. Masumoto S, Sato M, Ichinohe Y, Maeno T. Factors facilitating home death in non-cancer older patients receiving home medical care. Geriatrics & Gerontology International. 2019;19(12):1231-1235. | Population: dementia |
| 124. McCarthy EP, Burns RB, Davis RB, Phillips RS. Barriers to hospice care among older patients dying with lung and colorectal cancer. Journal of Clinical Oncology 2003; 21(4):728–35. Available from: URL: <Go to ISI>://WOS:000181002500025. | Population: not multimorbid |
| 125. McCarthy EP, Pencina MJ, Kelly-Hayes M, Evans JC, Oberacker EJ, D'Agostino RB et al. Advance Care Planning and Health Care Preferences of Community-Dwelling Elders: The Framingham Heart Study. Journals of Gerontology Series a-Biological Sciences and Medical Sciences 2008; 63(9):951–9. Available from: URL: <Go to ISI>://WOS:000260074800008. | Population: not multimorbid |
| 126. McCarthy EP, Phillips RS, Zhong ZS, Drews RE, Lynn J. Dying with cancer: Patients' function, symptoms, and care preferences as death approaches. Journal of the American Geriatrics Society 2000; 48(5):S110-S121. Available from: URL: <Go to ISI>://WOS:000087033000015. | Population: not multimorbid |
| 127. McDade-Montez E, Watson D, Beer A. Similarity, Agreement, and Assumed Similarity in Proxy End-of-Life Decision Making. Families Systems & Health 2013; 31(4):366–81. Available from: URL: <Go to ISI>://WOS:000329118600006. | Population: not multimorbid |
| 128. McMahan RD, Knight SJ, Fried TR, Sudore RL. Advance Care Planning Beyond Advance Directives: Perspectives From Patients and Surrogates. Journal of Pain and Symptom Management 2013; 46(3):355–65. Available from: URL: <Go to ISI>://WOS:000324026700005. | Population: not multimorbid |
| 129. McParland E, Likourezos A, Chichin E, Castor T, Paris BEC. Stability of preferences regarding life-sustaining treatment: A two-year prospective study of nursing home residents. Mount Sinai Journal of Medicine 2003; 70(2):85–92. Available from: URL: <Go to ISI>://WOS:000181351600003. | No full-text available |
| 130. Meads D, O'Dwyer J, Hulme C, Chintakayala P, Vinall-Collier K, Bennett M. Patient Preferences for Pain Management in Advanced Cancer: Results from a Discrete Choice Experiment. Patient 2017; 10(5):643–51. Available from: URL: http://www.redi-bw.de/db/ebsco.php/search.ebscohost.com/login.aspx%3fdirect%3dtrue%26db%3dcin20%26AN%3d125224004%26site%3dehost-live. | Population: not multimorbid |
| 131. Möller UO, Pranter C, Hagelin CL, et al. Using Cards to Facilitate Conversations About Wishes and Priorities of Patients in Palliative Care. J Hosp Palliat Nurs. 2020;22(1):33-39. | Population: not multimorbid |
| 132. Morgan DJR, Eng D, Higgs D, Beilin M, Bulsara C, Wong M et al. Advance care planning documentation strategies; goals-of-care as an alternative to not-for-resuscitation in medical and oncology patients. A pre-post controlled study on quantifiable outcomes. Internal Medicine Journal 2018; 48(12):1472–80. Available from: URL: http://ovidsp.ovid.com/ovidweb.cgi?T=JS&CSC=Y&NEWS=N&PAGE=fulltext&D=medl&AN=30043464. | Outcome: preferences not addressed |
| 133. Morton RL, Snelling P, Webster AC, Rose J, Masterson R, Johnson DW et al. Factors influencing patient choice of dialysis versus conservative care to treat end-stage kidney disease. CMAJ Canadian Medical Association Journal 2012; 184(5):E277-83. Available from: URL: http://ovidsp.ovid.com/ovidweb.cgi?T=JS&CSC=Y&NEWS=N&PAGE=fulltext&D=med8&AN=22311947. | Outcome: preferences not addressed |
| 134. Mukamel DB, Ladd H, Temkin-Greener H. Stability of Cardiopulmonary Resuscitation and Do-Not-Resuscitate Orders Among Long-term Nursing Home Residents. Medical Care 2013; 51(8):666–72. Available from: URL: <Go to ISI>://WOS:000323228500007. | Outcome: preferences from caregivers, family members and health care professionals |
| 135. Muller-Mundt G, Bleidorn J, Geiger K, Klindtworth K, Pleschberger S, Hummers-Pradier E et al. End of life care for frail older patients in family practice (ELFOP)--protocol of a longitudinal qualitative study on needs, appropriateness and utilisation of services. BMC Family Practice 2013; 14:52. Available from: URL: http://ovidsp.ovid.com/ovidweb.cgi?T=JS&CSC=Y&NEWS=N&PAGE=fulltext&D=med9&AN=23642254. | Outcome: preferences not addressed |
| 136. Murphy DJ, Burrows D, Santilli S, Kemp AW, Tenner S, Kreling B et al. THE INFLUENCE OF THE PROBABILITY OF SURVIVAL ON PATIENTS PREFERENCES REGARDING CARDIOPULMONARY-RESUSCITATION. New England Journal of Medicine 1994; 330(8):545–9. Available from: URL: <Go to ISI>://WOS:A1994MX43600007. | Population: not multimorbid |
| 137. Miyashita J, Kohno A, Cheng SY, et al. Patients' preferences and factors influencing initial advance care planning discussions' timing: A cross-cultural mixed-methods study. Palliat Med. 2020;34(7):906-916. | Population: not multimorbid |
| 138. Oosterveld-Vlug MG, Francke AL, Pasman HRW, Onwuteaka-Philipsen BD. How should realism and hope be combined in physician-patient communication at the end of life? An online focus-group study among participants with and without a Muslim background. Palliative & Supportive Care 2017; 15(3):359–68. Available from: URL: http://www.redi-bw.de/db/ebsco.php/search.ebscohost.com/login.aspx%3fdirect%3dtrue%26db%3dcin20%26AN%3d123303219%26site%3dehost-live. | Population: not multimorbid |
| 139. Overbeek A, Korfage IJ, Jabbarian LJ, Billekens P, Hammes BJ, Polinder S et al. Advance Care Planning in Frail Older Adults: a Cluster Randomized Controlled Trial. Journal of the American Geriatrics Society 2018; (no pagination). Available from: URL: https://www.cochranelibrary.com/central/doi/10.1002/central/CN-01572561/full. | Outcome: preferences not addressed |
| 140. Pardon K, Deschepper R, Vander Stichele R, Bernheim J, Mortier F, Deliens L. Preferences of advanced lung cancer patients for patient-centred information and decision-making: A prospective multicentre study in 13 hospitals in Belgium. Patient Education and Counseling 2009; 77(3):421–9. Available from: URL: http://www.redi-bw.de/db/ebsco.php/search.ebscohost.com/login.aspx%3fdirect%3dtrue%26db%3dpsyh%26AN%3d2009-19159-001%26site%3dehost-live. | Population: not multimorbid |
| 141. Patrick DL, Pearlman RA, Starks HE, Cain KC, Cole WG, Uhlmann RF. Validation of preferences for life-sustaining treatment: Implications for advance care planning. Annals of Internal Medicine 1997; 127(7):509-+. Available from: URL: <Go to ISI>://WOS:A1997XY73300002. | Population: not multimorbid |
| 142. Philippart F, Vesin A, Bruel C, Kpodji A, Durand-Gasselin B, Garcon P et al. The ETHICA study (part I): elderly's thoughts about intensive care unit admission for life-sustaining treatments. Intensive Care Medicine 2013; 39(9):1565–73. Available from: URL: <Go to ISI>://WOS:000322675500006. | Population: not multimorbid |
| 143. Piamjariyakul U, Myers S, Werkowitch M, Smith CE. End-of-life preferences and presence of advance directives among ethnic populations with severe chronic cardiovascular illnesses. European Journal of Cardiovascular Nursing 2014; 13(2):185–9. Available from: URL: <Go to ISI>://WOS:000332104200011. | Population: not multimorbid |
| 144. Pruchno RA, Lemay ER, Feild L, Levinsky NG. Predictors of patient treatment preferences and spouse substituted judgments: The case of dialysis continuation. Medical Decision Making 2006; 26(2):112–21. Available from: URL: <Go to ISI>://WOS:000236557400003. | Population: age < 60 yrs. |
| 145. Pruchno RA, Rovine MJ, Cartwright F, Wilson-Genderson M. Stability and change in patient preferences and spouse substituted judgments regarding dialysis continuation. Journals of Gerontology Series B-Psychological Sciences and Social Sciences 2008; 63(2):S81-S91. Available from: URL: <Go to ISI>://WOS:000255449800011. | Population: not multimorbid |
| 146. Raj R, Thiruvengadam S, Ahuja KDK, Frandsen M, Jose M. Discussions during shared decision-making in older adults with advanced renal disease: a scoping review. BMJ open. 2019;9(11):e031427-e031427. | Outcome: preferences not addressed |
| 147. Reeves MJ, Myers LJ, Williams LS, Phipps MS, Bravata DM. Do-not-resuscitate orders, quality of care, and outcomes in veterans with acute ischemic stroke. Neurology 2012; 79(19):1990–6. Available from: URL: http://www.redi-bw.de/db/ebsco.php/search.ebscohost.com/login.aspx%3fdirect%3dtrue%26db%3dpsyh%26AN%3d2012-33960-011%26site%3dehost-live. | Population: age < 60 yrs. |
| 148. Reilly BM, Magnussen CR, Ross J, Ash J, Papa L, Wagner M. CAN WE TALK - INPATIENT DISCUSSIONS ABOUT ADVANCE DIRECTIVES IN A COMMUNITY-HOSPITAL - ATTENDING PHYSICIANS ATTITUDES, THEIR INPATIENTS WISHES, AND REPORTED EXPERIENCE. Archives of Internal Medicine 1994; 154(20):2299–308. Available from: URL: <Go to ISI>://WOS:A1994PN18500004. | Population: not multimorbid |
| 149. Reinke LF, Slatore CG, Udris EM, Moss BR, Johnson EA, Au DH. The association of depression and preferences for life-sustaining treatments in veterans with chronic obstructive pulmonary disease. Journal of Pain and Symptom Management 2011; 41(2):402–11. Available from: URL: https://www.cochranelibrary.com/central/doi/10.1002/central/CN-00785684/full. | Population: not multimorbid |
| 150. Rodriguez Jornet A, Ibeas J, Real J, Pena S, Martinez Ocana JC, Garcia Garcia M. [Advance directives in chronic dialysis patients]. Nefrologia 2007; 27(5):581–92. Available from: URL: http://ovidsp.ovid.com/ovidweb.cgi?T=JS&CSC=Y&NEWS=N&PAGE=fulltext&D=med5&AN=18045034. | Population: not multimorbid |
| 151. Rosenfeld KE, Wenger NS, Phillips RS, Connors AF, Dawson NV, Layde P et al. Factors associated with change in resuscitation preference of seriously ill patients. Archives of Internal Medicine 1996; 156(14):1558–64. Available from: URL: <Go to ISI>://WOS:A1996UY33200009. | Outcome: preferences from caregivers, family members and health care professionals |
| 152. Rosenfeld KE, Wenger NS, Kagawa-Singer M. End-of-life decision making - A qualitative study of elderly individuals. Journal of General Internal Medicine 2000; 15(9):620–5. Available from: URL: <Go to ISI>://WOS:000089790900002. | Outcome: preferences not addressed |
| 153. Saeed F, Adams H, Epstein RM. Matters of Life and Death: Why Do Older Patients Choose Conservative Management? Am J Nephrol. 2020;51(1):35-42. | Outcome: preferences not addressed |
| 154. Sanchez-Tomero JA, Rodriguez-Jornet A, Balda S, Cigarran S, Herrero JC, Maduell F et al. Exploring the opinion of CKD patients on dialysis regarding end-of-life and Advance Care Planning. Nefrologia 2011; 31(4):449–56. Available from: URL: http://ovidsp.ovid.com/ovidweb.cgi?T=JS&CSC=Y&NEWS=N&PAGE=fulltext&D=med7&AN=21738248. | Population: not multimorbid |
| 155. Sanders JJ, Johnson KS, Cannady K, Paladino J, Ford DW, Block SD et al. From Barriers to Assets: Rethinking factors impacting advance care planning for African Americans. Palliative & Supportive Care 2019; 17(3):306–13. Available from: URL: <Go to ISI>://WOS:000474900300009. | Population: age < 60 yrs. |
| 156. Schamp R, Tenkku L. Managed death in a PACE: pathways in present and advance directives. Journal of the American Medical Directors Association 2006; 7(6):339–44. Available from: URL: http://www.redi-bw.de/db/ebsco.php/search.ebscohost.com/login.aspx%3fdirect%3dtrue%26db%3dcin20%26AN%3d106199330%26site%3dehost-live. | Outcome: preferences from caregivers, family members and health care professionals |
| 157. Schneiderman LJ, Pearlman RA, Kaplan RM, Anderson JP, Rosenberg EM. RELATIONSHIP OF GENERAL ADVANCE DIRECTIVE INSTRUCTIONS TO SPECIFIC LIFE-SUSTAINING TREATMENT PREFERENCES IN PATIENTS WITH SERIOUS ILLNESS. Archives of Internal Medicine 1992; 152(10):2114–22. Available from: URL: <Go to ISI>://WOS:A1992JT16300021. | Population: not multimorbid |
| 158. Schoenborn N, Janssen EM, Boyd CM, Bridges JFP, Wolff AC, Xue QL et al. Older adults' Communication preferences around discussing long-term life expectancy-results from a national survey. Journal of General Internal Medicine 2018; 33(2):274‐. Available from: URL: https://www.cochranelibrary.com/central/doi/10.1002/central/CN-01606167/full. | Population: not multimorbid |
| 159. Schonwetter RS, Walker RM, Solomon M, Indurkhya A, Robinson BE. Life values, resuscitation preferences, and the applicability of living wills in an older population. Journal of the American Geriatrics Society 1996; 44(8):954–8. Available from: URL: <Go to ISI>://WOS:A1996VA56300011. | Population: not multimorbid |
| 160. Schubart JR, Levi BH, Dellasega C, Whitehead M, Green MJ. Factors that affect decisions to receive (or not receive) life-sustaining treatment in advance care planning. Journal of Psychosocial Nursing & Mental Health Services 2014; 52(2):38–44. Available from: URL: http://ovidsp.ovid.com/ovidweb.cgi?T=JS&CSC=Y&NEWS=N&PAGE=fulltext&D=medc1&AN=24200911. | Population: not multimorbid |
| 161. Seah AST, Tan F, Srinivas S, Wu HY, Griva K. Opting out of dialysis - Exploring patients' decisions to forego dialysis in favour of conservative non-dialytic management for end-stage renal disease. Health Expectations 2015; 18(5):1018–29. Available from: URL: <Go to ISI>://WOS:000365046700033. | Outcome: preferences not addressed |
| 162. Seymour J, Gott M, Bellamy G, Ahmedzai SH, Clark D. Planning for the end of life: the views of older people about advance care statements. Social Science & Medicine 2004; 59(1):57–68. Available from: URL: <Go to ISI>://WOS:000221190600006. | Population: not multimorbid |
| 163. Shepardson LB, Gordon HS, Ibrahim SA, Harper DL, Rosenthal GE. Racial variation in the use of do-not-resuscitate orders. Journal of General Internal Medicine 1999; 14(1):15–20. Available from: URL: <Go to ISI>://WOS:000078157900003. | Population: not multimorbid |
| 164. Silveira MJ, DiPiero A, Gerrity MS, Feudtner C. Patients' knowledge of options at the end of life - Ignorance in the face of death. Jama-Journal of the American Medical Association 2000; 284(19):2483–8. Available from: URL: <Go to ISI>://WOS:000165183800022. | Outcome: preferences not addressed |
| 165. Singer PA, Thiel EC, Naylor CD, Richardson RM, Llewellyn-Thomas H, Goldstein M et al. Life-sustaining treatment preferences of hemodialysis patients: implications for advance directives. Journal of the american society of nephrology : JASN 1995; 6(5):1410–7. Available from: URL: https://www.cochranelibrary.com/central/doi/10.1002/central/CN-00123153/full. | Population: age < 60 yrs. |
| 166. Soskis CW. End-of-life decisions in the home care setting. Social Work in Health Care 1997; 25(3):107–16. Available from: URL: http://www.redi-bw.de/db/ebsco.php/search.ebscohost.com/login.aspx%3fdirect%3dtrue%26db%3dcin20%26AN%3d107257635%26site%3dehost-live. | Population: not multimorbid |
| 167. Stapleton RD, Nielsen EL, Engelberg RA, Patrick DL, Curtis JR. Ethics in cardiopulmonary medicine - Association of depression and life-sustaining treatment preferences in patients with COPD. Chest 2005; 127(1):328–34. Available from: URL: <Go to ISI>://WOS:000226365200051. | Outcome: preferences not addressed |
| 168. Talabani N, Ängerud KH, Boman K, Brännström M. Patients' experiences of person-centred integrated heart failure care and palliative care at home: an interview study. BMJ Support Palliat Care. 2020;10(1):e9. | Outcome: preferences not addressed |
| 169. Tang ST, Liu TW, Lai MS, Liu LN, Chen CH. Concordance of preferences for end-of-life care between terminally ill cancer patients and their family caregivers in Taiwan. Journal of Pain and Symptom Management 2005; 30(6):510–8. Available from: URL: <Go to ISI>://WOS:000234605300004. | Population: not multimorbid |
| 170. Tang ST, Wen FH, Hsieh CH, Chou WC, Chang WC, Chen JS et al. Preferences for Life-Sustaining Treatments and Associations With Accurate Prognostic Awareness and Depressive Symptoms in Terminally Ill Cancer Patients' Last Year of Life. Journal of Pain and Symptom Management 2016; 51(1):41–51. Available from: URL: <Go to ISI>://WOS:000367379000008. | Population: not multimorbid |
| 171. Tang ST, Wen FH, Chang WC, Hsieh CH, Chou WC, Chen JS et al. Preferences for Life-Sustaining Treatments Examined by Hidden Markov Modeling Are Mostly Stable in Terminally Ill Cancer Patients' Last Six Months of Life. Journal of Pain and Symptom Management 2017; 54(5):628-+. Available from: URL: <Go to ISI>://WOS:000414744700003. | Population: not multimorbid |
| 172. Thompson S, Lyford M, Papertalk L, Holloway M. Passing on wisdom: exploring the end-of-life wishes of Aboriginal people from the Midwest of Western Australia. Rural Remote Health. 2019;19(4):5444. | Population: not multimorbid |
| 173. Towsley GL, Hirschman KB. Talking about end-of-life care: Perspectives of nursing home residents. Geriatric Nursing 2018; 39(1):72–6. Available from: URL: <Go to ISI>://WOS:000428971600011. | Population: not multimorbid |
| 174. Towsley GL, Hirschman KB, Madden C. Conversations about End of Life: Perspectives of Nursing Home Residents, Family, and Staff. Journal of Palliative Medicine 2015; 18(5):421–8. Available from: URL: <Go to ISI>://WOS:000353710300008. | Outcome: preferences from caregivers, family members and health care professionals |
| 175. Tsevat J, Cook EF, Green ML, Matchar DB, Dawson NV, Broste SK et al. HEALTH VALUES OF THE SERIOUSLY ILL. Annals of Internal Medicine 1995; 122(7):514–20. Available from: URL: <Go to ISI>://WOS:A1995QP00800007. | Population: not multimorbid |
| 176. Tsevat J, Dawson NV, Wu AW, Lynn J, Soukup JR, Cook EF et al. Health values of hospitalized patients 80 years or older. Jama-Journal of the American Medical Association 1998; 279(5):371–5. Available from: URL: <Go to ISI>://WOS:000071731300027. | Population: not multimorbid |
| 177. Uhlmann RF, Pearlman RA. PERCEIVED QUALITY-OF-LIFE AND PREFERENCES FOR LIFE-SUSTAINING TREATMENT IN OLDER ADULTS. Archives of Internal Medicine 1991; 151(3):495–7. Available from: URL: <Go to ISI>://WOS:A1991FA76800010. | No full-text available |
| 178. Umezawa S, Fujimori M, Matsushima E, Kinoshita H, Uchitomi Y. Preferences of advanced cancer patients for communication on anticancer treatment cessation and the transition to palliative care. Cancer 2015; 121(23):4240–9. Available from: URL: http://www.redi-bw.de/db/ebsco.php/search.ebscohost.com/login.aspx%3fdirect%3dtrue%26db%3dpsyh%26AN%3d2015-53698-017%26site%3dehost-live. | Population: not multimorbid |
| 179. van Wijmen MPS, Pasman HRW, Twisk JWR, Widdershoven GAM, Onwuteaka-Philipsen BD. Stability of end-of-life preferences in relation to health status and life-events: A cohort study with a 6-year follow-up among holders of an advance directive. Plos One 2018; 13(12). Available from: URL: <Go to ISI>://WOS:000453774100048. | Population: not multimorbid |
| 180. Vig EK, Pearlman RA. Good and bad dying from the perspective of terminally ill men. Archives of Internal Medicine 2004; 164(9):977–81. Available from: URL: <Go to ISI>://WOS:000221277100008. | Outcome: preferences not addressed |
| 181. Visser A, Dijkstra GJ, Kuiper D, Jong PE de, Franssen CF, Gansevoort RT et al. Accepting or declining dialysis: considerations taken into account by elderly patients with end-stage renal disease. Journal of Nephrology 2009; 22(6):794–9. Available from: URL: http://ovidsp.ovid.com/ovidweb.cgi?T=JS&CSC=Y&NEWS=N&PAGE=fulltext&D=med6&AN=19967659. | Outcome: preferences not addressed |
| 182. Weeks JC, Cook EF, O'Day SJ, Petersen LM, Wenger N, Reding D et al. Relationship between cancer patients' predictions of prognosis and their treatment preferences. Jama-Journal of the American Medical Association 1998; 279(21):1709–14. Available from: URL: <Go to ISI>://WOS:000073878700031. | Population: not multimorbid |
| 183. Wen FH, Chen JS, Chou WC, Chang WC, Hsieh CH, Tang ST. Extent and Determinants of Terminally Ill Cancer Patients' Concordance Between Preferred and Received Life-Sustaining Treatment States: An Advance Care Planning Randomized Trial in Taiwan. Journal of Pain and Symptom Management 2019; 58(1):1-+. Available from: URL: <Go to ISI>://WOS:000472494200006. | Population: not multimorbid |
| 184. Winter L. Patient Values and Preferences for End-of-Life Treatments: Are Values Better Predictors Than a Living Will? Journal of Palliative Medicine 2013; 16(4):362–8. Available from: URL: <Go to ISI>://WOS:000316857000010. | Outcome: preferences not addressed |
| 185. Winter L, Lawton MP, Ruckdeschel K. Preferences for prolonging life: a prospect theory approach. International Journal of Aging & Human Development 2003; 56(2):155–70. Available from: URL: http://ovidsp.ovid.com/ovidweb.cgi?T=JS&CSC=Y&NEWS=N&PAGE=fulltext&D=med4&AN=14533855. | Population: not multimorbid |
| 186. Wittink MN, Morales KH, Meoni LA, Ford DE, Wang NY, Klag MJ et al. Stability of Preferences for End-of-Life Treatment After 3 Years of Follow-up - The John Hopkins Precursors Study. Archives of Internal Medicine 2008; 168(19):2125–30. Available from: URL: <Go to ISI>://WOS:000260332400010. | Outcome: preferences from caregivers, family members and health care professionals |
| 187. Wright AA, Mack JW, Kritek PA, Balboni TA, Massaro AF, Matulonis UA et al. Influence of patients' preferences and treatment site on cancer patients' end-of-life care. Cancer 2010; 116(19):4656–63. Available from: URL: http://www.redi-bw.de/db/ebsco.php/search.ebscohost.com/login.aspx%3fdirect%3dtrue%26db%3dpsyh%26AN%3d2010-19790-002%26site%3dehost-live. | Population: age < 60 yrs. |
| 188. Young KA, Wordingham SE, Strand JJ, Roger VL, Dunlay SM. Discordance of Patient-Reported and Clinician-Ordered Resuscitation Status in Patients Hospitalized With Acute Decompensated Heart Failure. Journal of Pain & Symptom Management 2017; 53(4):745–50. Available from: URL: http://www.redi-bw.de/db/ebsco.php/search.ebscohost.com/login.aspx%3fdirect%3dtrue%26db%3dcin20%26AN%3d122085100%26site%3dehost-live. | Outcome: preferences from caregivers, family members and health care professionals |
| 189. Zafar SY, Malin JL, Grambow SC, Abbott DH, Kolimaga JT, Zullig LL et al. Chemotherapy Use and Patient Treatment Preferences in Advanced Colorectal Cancer A Prospective Cohort Study. Cancer 2013; 119(4):854–62. Available from: URL: <Go to ISI>://WOS:000314657000022. | Population: not multimorbid |
| 190. Zwakman M, Kars MC, Caswell G, Christensen CA, Deliens L, Ingravallo F et al. Content analysis of the action advance care planning document completed by patients with advanced cancer: insights gained from the action trial. Palliative Medicine 2018; 32(1):158–9. Available from: URL: https://www.cochranelibrary.com/central/doi/10.1002/central/CN-01606096/full. | Population: not multimorbid |
