# Supplementary material for: End-of-Life Care Preferences of Older Patients with Multimorbidity: A Mixed Methods Systematic Review
Source: J Clin Med. 2020 Dec 29;10(1):91. doi: 10.3390/jcm10010091 (PMC7795676; doi:10.3390/jcm10010091)
Supplement: Supplementary file 1 [file jcm-10-00091-s001.zip › jcm-1043522-supplementary/Supplementary Figure 1_PRISMA 2009 flow diagram.doc]

**PRISMA 2009 Flow Diagram**

**Screening**

**Included**

**Eligibility**

**Identification**

Records identified through database searching
(n = 5,255)

Additional records identified through other sources
(n = 262)

Records after duplicates removed
(n = 490)

Records screened
(n = 5,027)

Records excluded
(n = 4,815)

Full-text articles assessed for eligibility
(n = 212)

Studies included in qualitative synthesis
(n = 22)

Studies included in quantitative synthesis (meta-analysis)
(n = 14)

Full-text articles excluded, with reasons
(n = 190)

-Outcome: End-of-life care preferences not addressed (n = 49)

-Outcome: Preferences from physicians, caregivers… (n= 19)

-Population: Not multimorbid (n = 98)

-Population: Age < 60 yrs. (n = 12)

-Population: Dementia (n = 1)

-Document type: Editorials, narrative reviews (n = 2)

-Document type: scoping or systematic reviews (n = 7)

-No full-text available (n=2)
